# Designing Catalysts to Accelerate a Protein–Peptide Assembly-Reaction Cascade

**DOI:** 10.1021/acscentsci.5c00481

**Published:** 2025-06-16

**Authors:** Yibin Sun, Yue Fang, Yajie Liu, Fengyi Jiang, Zhichao Lei, Hanyu Gao, Wen-Bin Zhang

**Affiliations:** ‡ Department of Polymer Science and Engineering, College of Chemistry and Molecular Engineering, 12465 Peking University, Beijing 100871, People’s Republic of China; § Beijing National Laboratory for Molecular Sciences, Key Laboratory of Polymer Chemistry & Physics of Ministry of Education, Center for Soft Matter Science and Engineering, Peking University, Beijing 100871, People’s Republic of China; ¶ Department of Chemical and Biological Engineering, 58207 Hong Kong University of Science and Technology, Clear Water Bay, Hong Kong SAR, People’s Republic of China; # Artificial Intelligence for Science-Preferred Program, Shenzhen Graduate School, Peking University, Shenzhen 518055, People’s Republic of China

## Abstract

A biological system is rich in dynamic biomolecular assembly reaction cascades mediated by enzymes and molecular chaperones, as represented by the formation of the “chain-mail-like” bacteriophage HK97 capsid that involves sequential events of chaperone-assisted assembly and cross-linking reactions. To shed light on such catalyzed assembly processes, we report an artificial protein–peptide “assembly-reaction” cascade that can be accelerated by rationally designed catalysts. The cascade is inhibited by a tethered SpyTag mutant that blocks SpyCatcher from the subsequent reactions. A designed fusion of calmodulin and sortase can promote the cascade by first binding with M13 at the loop between the SpyTag mutant and SpyCatcher to open the gating via a coil–helix transition. After the SpyTag-SpyCatcher reaction, the catalyst is regenerated by sortase-mediated cyclization that restores the constrained M13 conformation at the loop to release the bounded calmodulin. In the presence of 0.1 equiv of catalyst, the process can be accelerated, increasing the initial rate by ∼12-fold and reducing the half-life by ∼17-fold. With experimentally measured kinetic parameters, we simulated this system through microkinetic modeling, illustrated the contributions of each parameter, and proposed conditions for optimal catalytic performance. As a prototype of artificial catalyzed supramolecular “assembly-reaction” cascades, this work is reminiscent of those catalyzed cascades in nature. Their common features reveal similar underlying physiochemical principles and suggest new avenues to understand and interfere with biological systems.

## Graphical abstract

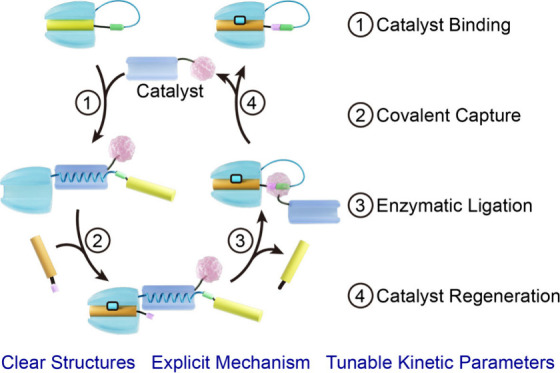

## Introduction

A biological system is a complex network of molecular assembly and reactions that controls the flow and interconversion of matters and energy.^1–5^ Assembly occurs constantly from folding of a polypeptide chain to maturation of a delicate molecular machine, which is often coupled with reactions to steer the direction of an “assembly-reaction” cascade.^6–10^ Within the complex cellular environment, such a process is often complicated by competing pathways, or steps with high kinetic barrier, or steps that require spatiotemporal control.^11–14^ Nature addresses this problem by molecular chaperones that can catalytically alter the pathway or accelerate the assembly/reaction cascade as shown in the assisted folding of nascent peptide chains,^15–19^ the dynamic assembly/disassembly of ribosomes and nucleosome,^20–22^ among others.^23^ This has enabled delicate functions like signal amplification, metabolic turnover, etc.^6^ Despite the prevalence of catalyzed assembly reaction cascades in living organisms, we still have a rather limited understanding on their molecular mechanisms.^23^

In fact, nature’s pervasive examples of catalyzed assembly reaction cascades have inspired chemists to create rationally designed catalytically controlled supramolecular systems.^24^ To date, the development of generalizable catalytic cascades with quantitative control has been realized in only very limited cases,^24–31^ mostly on small molecules or nucleic acids.^32–36^ Nonetheless, constructing these systems remains profoundly challenging as beneficial factors for one elementary step often inhibit another (e.g., catalyst regeneration), particularly in systems approaching thermodynamic equilibrium. For example, chiral barbiturates are reported to catalyze the interconversion of supramolecular enantiomers,^25^ chiral organic cages are found to catalyze supramolecular polymerization with an enhanced rate and product enantioselectivity,^26^ and the electron is shown to catalyze the formation of a trisradical complex.^27^ It is highly desired to develop a model assembly reaction system that can be accelerated by a rationally designed catalyst with an explicit mechanism. Such systems can shed light on the thermodynamic and kinetic features of catalyzed assembly processes in general and improve our understanding of similar biological systems. We reasoned that biomolecular interaction modules, such as the noncovalent interaction between calmodulin (CaM) and its binding peptide (such as the M13 peptide) forming a 1:1 CaM·M13 complex, and the covalent SpyCatcher-SpyTag reaction (Scheme 1a–c),^37–41^ are ideal candidates for this purpose because they are already used in biological systems and known for their versatile structures, programmable affinities, and implicit conformational changes.^42–48^

**Scheme 1:**
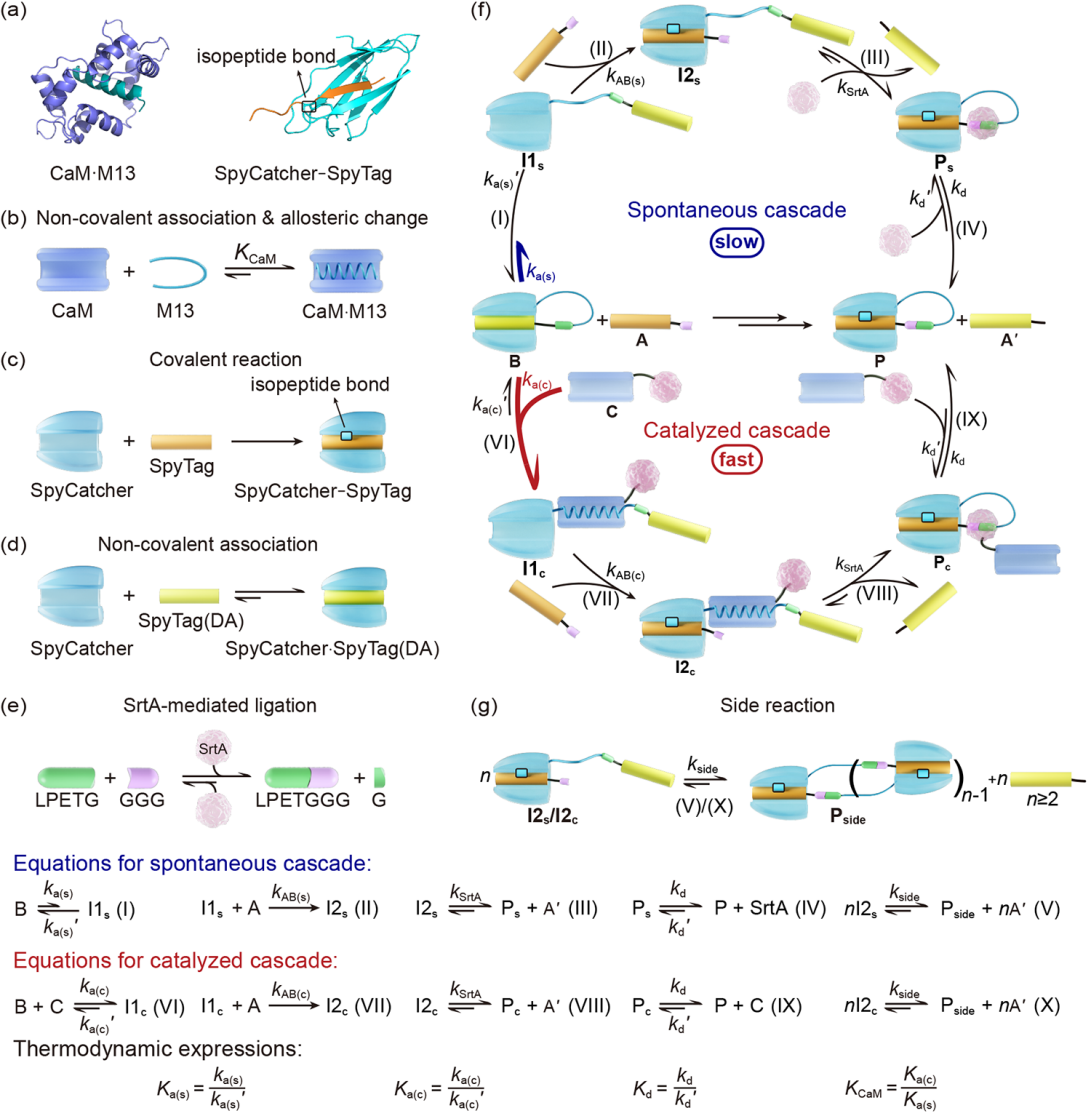
A Model Assembly-Reaction Cascade That Can Be Accelerated by a Rationally Designed Catalyst^a^

a (a) Structures of the noncovalent complex, CaM·M13 (PDB code: 1QTX; CaM: purple; M13: green), and the covalent complex, SpyCatcher-SpyTag (PDB code: 4MLI; SpyTag: orange; SpyCatcher: cyan). Schematic illustration of (b) the noncovalent binding between CaM and M13 & the conformational change of M13 upon association; (c) the covalent reaction between SpyCatcher and SpyTag; (d) the noncovalent association between SpyCatcher and SpyTag­(DA); (e) the SrtA-mediated ligation; (f) the designed spontaneous cascade and catalyzed cascade; and (g) the side reaction.

Herein, we report a model assembly reaction cascade that can be accelerated by rationally designed catalysts using the two motifs mentioned above (Scheme 1a–d) and an enzyme-mediated ligation (Scheme 1e). Not only can the conformation of the M13 segment—whose structural transition is closely coupled to catalyst binding and regeneration—be reliably predicted using AlphaFold2,^49^ which supports the hypothetical gating mechanism during the cascade, but also the reaction kinetics can be precisely and comprehensively simulated by microkinetic modeling.^50–52^ Notably, this genetically programmable system also provides further opportunities for fine-tuning, systematic exploration, and directed evolution of catalytic performance. To the best of our knowledge, this is the first example of a genetically encoded, rationally designed catalyst that accelerates an artificial assembly reaction cascade through conformational changes and covalent transformation, providing a biologically inspired model system with an explicit mechanism.

## Results and Discussion

### Cascade Design and Experimental Validation

The cascade design is shown in Scheme 1f with substrates **A** (GGG-SpyTag, or SpyTag with a GGG sequence at the N-terminus) and **B** (SpyCatcher-M13-LPETG-SpyTag­(DA)). SpyCatcher is known to form an isopeptide bond with SpyTag, but only forms a noncovalent complex with the nonreactive Asp117Ala (DA) mutant of SpyTag, or SpyTag­(DA).^38,53^ In this study, SpyTag­(DA) specifically refers to the SpyTag002­(DA) variant,^39^ which exhibits tighter binding to SpyCatcher and slower displacement kinetics than the DA mutant of the first-generation, wild-type SpyTag. The disordered M13 peptide at the loop region in **B** allows SpyTag­(DA) to block the active site of SpyCatcher via noncovalent association, which reduces the concentration of intermediate **I1**_**s**_ and slows down the ligation between **A** and **B**. Subsequently, the transpeptidase, sortase A (SrtA),^54–56^ recognizes the LPET/G sequence in **B**, cleaves between T (Thr) and G (Gly), and links T to the N-terminal glycine oligomer (such as GGG) of **A** to give a tadpole protein **P**, as the final product. The overall rate of the cascade will be slow, as SpyTag­(DA) noncovalently occupies the binding pocket of SpyCatcher and its spontaneous dissociation and subsequent replacement by reactive SpyTag will be delayed.

To accelerate the cascade, we designed fusion protein **C** (CaM-SrtA) as the catalyst. We hypothesized that the association of CaM with M13 peptide in **B** triggers the coil–helix transition of M13 peptide at the loop region,^42–45^ which shall open the gating and promote the cascade. After the SpyTag-SpyCatcher reaction, SrtA restores the loop between SpyCatcher and SpyTag and resets the M13 peptide to the disordered loop conformation. This conformational change discourages binding with CaM, leading to its spontaneous dissociation. The catalyst regeneration thus fulfills the cycle (see Supporting Information section Additional Discussions Part A for more related discussions). The entire process is further facilitated by the spatial proximity of CaM and SrtA in the fusion. This is thus referred to as the catalyzed cascade (AlphaFold2 predicted structures are shown in Figure S1).

We thus proceeded with experimental validation. For a better resolution in sodium dodecyl sulfate-polyacrylamide gel electrophoresis (SDS-PAGE) analysis, a yellow fluorescent protein (YFP) is fused at the C-terminus of substrate **A** (GGG-SpyTag-YFP), but omitted in our maintext for clarity. Both substrates **A** and **B** were recombinantly expressed in *Escherichia coli* and purified using affinity chromatography. They were mixed in a 1:1 ratio with 0.1 equiv of SrtA or **C** in Tris-HCl buffer. The kinetics was followed by SDS-PAGE (Figure 1a) and analyzed by densitometry (Figure 1b). The product was further confirmed by LC-MS (Figure 1c). As expected, the rate was accelerated with only 0.1 equiv of **C**, increasing the yield at 8 h from ∼10% without catalyst to ∼56% with catalyst. No intermediates (**I2**_**s**_ or **I2**_**c**_) were observed in the LC-MS spectra. The complete cyclization indicates that SrtA was sufficiently fast and largely irreversible in both cascades. This is consistent with the reported very high kinetics of *k*_cat_ = 5.4 s^–1^ and *k*_cat_/*K*_m_ = 23000 M^–1^ s^–1^ for the SrtA-5M^55^ that we used. To examine the reversibility of the SrtA-mediated cyclization in this cascade, we mixed the product with SrtA at a 1:0.1 and 1:1 molar ratio for extended times and analyzed the mixture by LC-MS. The results show no signal of the cleaved product over 16 h, indicating its intact structure (see Supporting Information section Additional Discussions Part B for details). We thus conclude that while the inherent reversibility of the SrtA-mediated ligation may still play a minor role, the cyclization is nonetheless practically irreversible.

**Figure 1:**
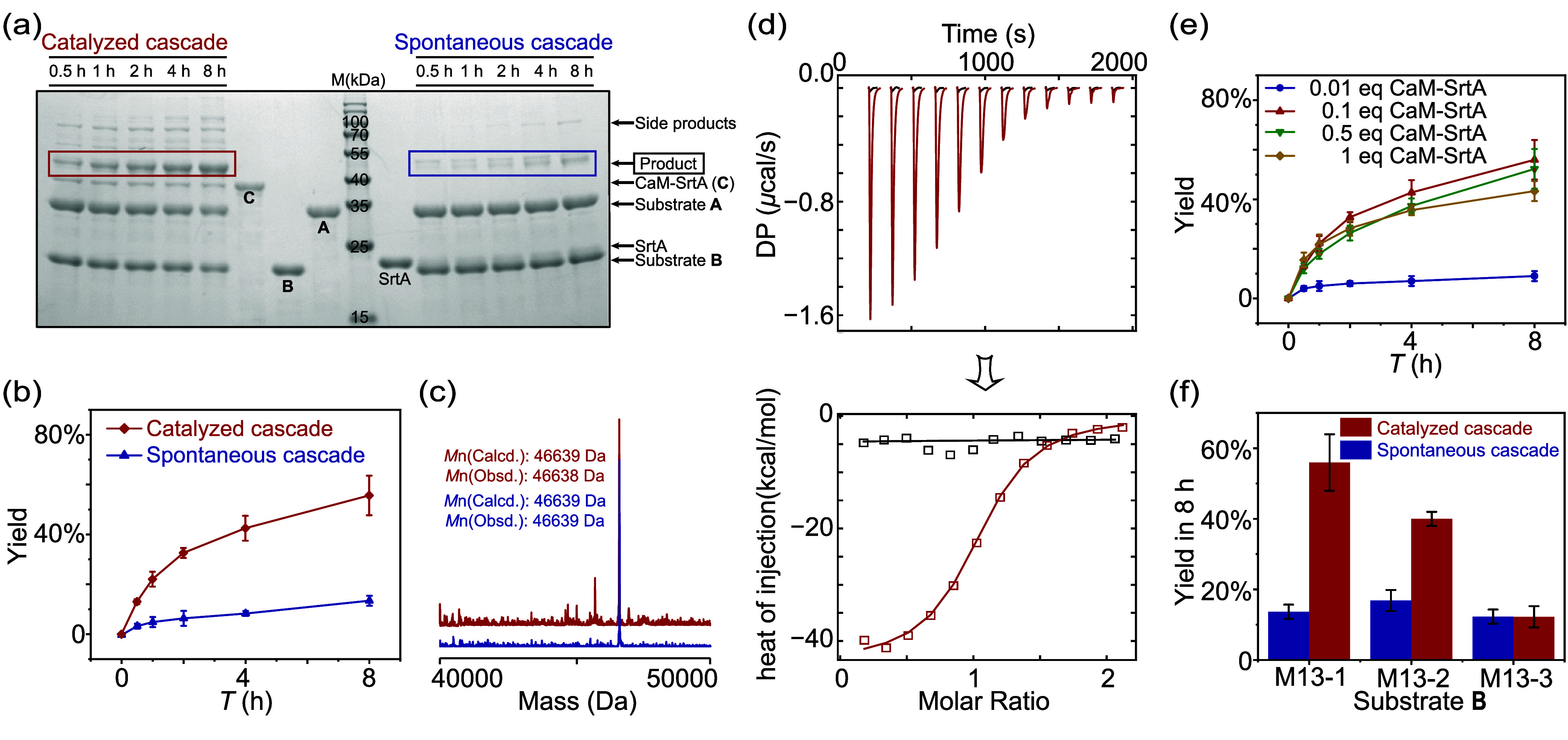
Accelerating the assembly reaction cascade with a catalyst, **C** (CaM-SrtA). (a) Investigating the kinetics of a catalyzed cascade (with 0.1 equiv of **C**) and spontaneous cascade (with 0.1 equiv of SrtA) by Coomassie-stained SDS-PAGE (cropped gel). An uncropped gel is shown in Figure S5c. (b) Kinetic profiles of yield versus time (*T*) for catalyzed (red) and spontaneous (blue) cascades. (c) MS spectra of the products obtained by catalyzed (red) and spontaneous (blue) cascades. (d) Isothermal titration thermograms of CaM (200 μM) titrated into **B** (18 μM, red) and **P** (18 μM, black). (e) Kinetic profiles of the stoichiometry-dependent study on **C**. (f) M13-dependent yield of catalyzed (red) and spontaneous (blue) cascades in 8 h. In all cases, **B** refers to M13-1 unless indicated otherwise. All kinetic experiments are carried out at [A]_0_ = [B]_0_ = 10 μM, Tris-HCl buffer, pH = 8.0, 10 mM CaCl_2_, 37 °C; error bars are based on three replicates.

Considering that the reverse cleavage reaction can be suppressed under certain conditions such as inducing β-hairpin^57^ and cyclic^58^ structures, the apparent irreversibility is presumably due to the fact that the recognition sequence in **P**_**c**_ or **P**_**s**_ resides in the loop and is conformationally restricted as compared to the precursor (**I2**_**c**_ or **I2**_**s**_) with free ends, leading to much lower binding affinity with SrtA. Hence, the reverse reaction is much slower than the forward reaction, making it virtually irreversible. The absence of product cleavage also implies the efficient catalyst regeneration in the catalyzed cascade. To support the spontaneous catalyst regeneration, we determined the binding constants of CaM to **B** and **P** by isothermal titration calorimetry (ITC, Figure 1d and Table S1). CaM has a high binding affinity to **B** (*K*_a(c)_ = 4.8 × 10^5^ M^–1^), but not to **P** (undetectable, typically <100 M^–1^).^59,60^ The dramatic difference thus underlies the spontaneous release of the catalyst from the product. We have not determined the binding constants of SrtA to **B** and **P** by ITC, because this process involves also chemical transformation that are hard to decouple. However, the physical picture is quite similar. The results establish a model catalyzed cascade based on well-known peptide–protein interactions.

We also examined the dependence of the yield on catalyst loading (Figure 1e). Surprisingly, the yield in 8 h is optimal at 0.1 equiv, while a stoichiometric amount of catalyst leads to a decrease in yield despite higher conversion of substrates. We attribute this to intermolecular side reactions that give high-molecular-weight products, as seen in SDS-PAGE (Figure S2). We also recognize that although it is possible for SrtA to cleave SpyTag­(DA) before its disassociation from SpyCatcher (Figure S3), this is unlikely to be significant at low catalyst loading since the recognition sequence for SrtA also adopts a highly constrained conformation in the loop and becomes hard to bind. However, this may be more severe at high catalyst loading when more SrtA are available.

As a model system, we can also independently vary the binding affinity between the catalyst and substrate to tune the catalytic performance. We replaced the original M13 (named as M13-1) in **B** with two designed M13 variants, M13-2 (*K*_a(c)_ = 2.0 × 10^4^ M^–1^) and M13-3 (undetectable, typically <100 M^–1^) (Figure S4 and Table S1). As expected, rate acceleration was still observed for M13-2, but not for M13-3 (Figure 1f and Figures S5–S7). This model system thus offers an opportunity to quantitatively look into how catalysts work on an assembly reaction cascade.

### Kinetic Analysis

Based on the designed mechanism, we used both conventional rate-determining approximation and the all-parameter microkinetic modeling^50,51^ to analyze the kinetics. The former with a simplified physical picture reveals an initial reaction rate enhancement by ∼12-fold and a decrease in half-life by ∼17-fold (see Supporting Information section Kinetic Analysis Using Rate-Determining Approximation for details). While the kinetics of both SrtA-mediated ligation and SpyTag-SpyCatcher reaction can vary over a very broad range, we assume that by design the SrtA-mediated ligation should be much faster than the SpyTag-SpyCatcher reaction to ensure efficient catalyst regeneration for a remarkable catalytic performance. As discussed previously, we used a very efficient SrtA mutant, the SrtA-5 M with *k*_cat_ = 5.4 s^–1^ and *k*_cat_/*K*_m_ = 23000 M^–1^ s^–1^, and the first generation of SpyTag-SpyCatcher reaction, with a measured *k*_AB_ ∼ 90 M^–1^ s^–1^ in our experiments. The distinct kinetics naturally makes the SpyTag-SpyCatcher reaction the rate-limiting step. This is also supported by the absence of the intermediates (**I2**_**c**_ or **I2**_**s**_) in our characterization. The rate-limiting nature of SpyTag-SpyCatcher ligation (low *k*_AB_) is consistent with the catalytic behavior, as it acts as a single, shared bottleneck in both cascades to control turnover, allowing the upstream gating module to meaningfully influence downstream reaction flux.

As for the all-parameter microkinetic modeling, it allows for comprehensive simulation and sensitivity analysis of all important parameters. Although SrtA-mediated ligation is inherently reversible, it was treated as a first-order irreversible reaction in our simulation with a composite, effective rate constant *k*_SrtA_ that takes the binding of SrtA and **B**, the catalytic ligation, and the very minor (if possible) reversible reaction all into consideration for simplicity. As we discussed in a previous section, this should be a reasonable hypothesis. In addition to the steps described in Scheme 1f, we also consider all the possible side reactions as a simplified composite reaction (Scheme 1g) in first order kinetics with a composite, effective rate constant, *k*_Side_. All of the reaction equations are shown in Scheme 1 (bottom), and the reaction rates can thus be described using the formulas as shown in Figures S8–S9. The microkinetic model was then developed and executed in a Jupyter Notebook, and fitted with experimental data ([A]_0_ = [B]_0_ = 10 μM, M13-1) to obtain the key parameters as shown in Table S1 (see the Supporting Information section Supplementary Methods for details). Because [P_c_] and [P_s_] could not be distinguished by SDS-PAGE, they are collectively counted as the final product. The simulated kinetic profiles match well with the experimental data (Figure 2a). The striking difference between *K*_a(c)_ (4.8 × 10^5^ M^–1^) and *K*_a(s)_ (0.01) underlies their distinct kinetics. This large binding affinity difference ensures that the “gate” is virtually closed in the absence of catalyst and effectively opened upon a CaM-triggered conformational change, which is a prerequisite for catalytic acceleration. This low equilibrium constant (*K*_a(s)_ = 0.01) in the spontaneous cascade leads to a lower effective concentration of **I1**_**s**_, prohibiting the subsequent rate-determining step. In contrast, the catalyzed cascade features a profound “gate-opening” event (*K*_a(c)_ = 4.8 × 10^5^ M^–1^) with much a higher effective concentration of **I1**_**c**_ to promote the reaction and accelerate the overall rate of the cascade. Together, these kinetic parameters reinforce the conformational gating mechanism, ensuring that substrate **A** effectively enters the SpyCatcher cavity in the catalyzed cascade.

**Figure 2:**
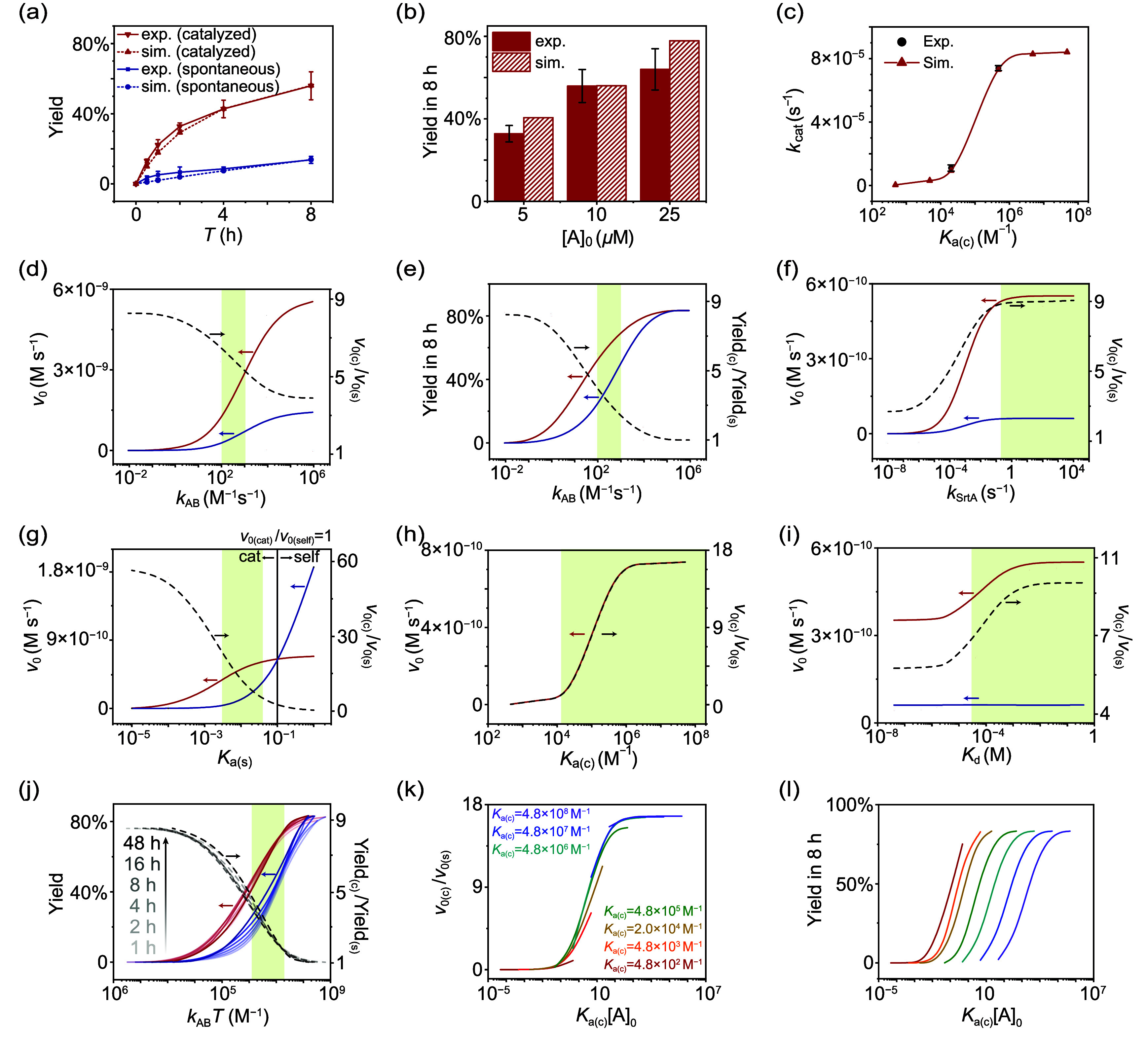
Kinetic analysis via microkinetic modeling and comprehensive sensitivity analysis. (a) Experimentally obtained (solid line) and simulated (dashed line) kinetic profiles of catalyzed (red) and spontaneous (blue) cascades. (b) Experimentally obtained (solid) and simulated (dashed) results of yield in 8 h with different [A]_0_. (c) Experimentally obtained (black) and simulated (red) profiles of *k*_cat_ (rate constant of catalyzed cascade using first order kinetic model) *versus K*_a(c)_. Simulated profiles of (d) initial rate (*v*_0_) *versus k*_AB_ for both catalyzed and spontaneous cascades (solid line), and the corresponding initial rate enhancement (*v*_0(c)_/*v*_0(s)_, dashed line); (e) yield in 8 h *versus k*_AB_ for both catalyzed and spontaneous cascades (solid line), and the corresponding yield gain (Yield_(c)_/Yield_(s)_) in 8 h (dashed line); (f) *v*_0_ *versus k*_SrtA_ for both catalyzed and spontaneous cascades­(solid line), and the corresponding *v*_0(c)_/*v*_0(s)_ (dashed line); (g) *v*_0_ *versus K*_a(s)_ (*K*_CaM_ = 4.8 × 10^7^ M^–1^) for both catalyzed and spontaneous cascades (solid line), and the corresponding *v*_0(c)_/*v*_0(s)_ (dashed line); as the apparent binding constant *K*_a(c)_ is governed by sequential SpyCatcher-SpyTag­(DA) dissociation (*K*_a(s)_) followed by CaM-M13 binding, *K*_a(s)_ directly influences the overall kinetics of catalyzed cascade; (h) *v*_0_ *versus K*_a(c)_ (*K*_a(s)_ = 0.01) for catalyzed cascade (solid line), and the corresponding *v*_0(c)_/*v*_0(s)_ (dashed line); (i) *v*_0_ *versus K*_d_ for both catalyzed and spontaneous cascades (solid line), and the corresponding *v*_0(c)_/*v*_0(s)_ (dashed line); (j) yield *versus k*_AB_ *T* for both catalyzed and spontaneous cascades (solid line) at various times, and the corresponding Yield_(c)_/Yield_(s)_ (dashed line); (k) *v*_0(c)_/*v*_0(s)_ *versus K*_a(c)_[A]_0_ at various *K*_a(c)_; and (i) yield in 8 h *versus K*_a(c)_[A]_0_ for catalyzed cascade. Catalyzed cascade are labeled in red, and spontaneous cascades are labeled in blue for all the profiles in (d)–(j). All simulations were carried out at [A]_0_ = [B]_0_ = 10 μM in the presence of 0.1 equiv of **C**, and the parameters employed are those outlined in Table 1 unless indicated otherwise; error bars are based on three replicates; arrows point toward the respective vertical axis (left or right) for each data curve.

Notably, *k*_SrtA_ can be simulated at any values not less than 10 s^–1^, proving our conjecture that SrtA-mediated ligation is a fast step in both cascades. As the side reaction is mainly due to intermolecular coupling via SrtA-mediated ligation, *k*_side_ is simulated as a *k*_SrtA_-dependent value (∼0.12 *k*_SrtA_), which produces ∼10% side products at 0.1 equiv of **C** or SrtA. The simulated reaction profiles also show excellent agreement with other experimental settings ([A]_0_ = [B]_0_ = 5 μM & 25 μM for M13-1 in Figure 2b, and [A]_0_ = [B]_0_ = 10 μM for M13-2 & M13-3 in Figure 2c and Figure S10). The high reliability of the microkinetic modeling allows comprehensive sensitivity analysis by varying the parameters over a broad range, especially those shared by both cascades such as *k*_AB_, *k*_SrtA_, and *K*_d_. Additionally, it also enables the prediction of the time-dependent concentration change of intermediates (Figure S11).

To facilitate the comparison between two cascades, we assume that they share the same values of *K*_d_, *k*_SrtA_, and *k*_Side_. In reality, their detailed values may be slightly different yet largely on the same scale in two cascades, as reflected in the values of *k*_AB_ (67 vs 90 M^–1^s^–1^). It is convenient to use the same set of values in simulation for comparison, from which we believe the basic conclusion about the trending should be as robust and helpful for guiding experimental optimization. The basic parameters were set as those in Table 1 unless otherwise noted.

**Table 1:** Fitted Parameters for the Microkinetic Model

	spontaneous	catalyzed
*k*_a(c)_ (M^–1^ s^–1^)^a^	–	4.8 × 10^3^
*k*_a(c)_′ (s^–1^)^a^	–	0.01
*K*_a(c)_ (M^–1^)^b^	–	4.8 × 10^5^
*k*_a(s)_ (s^–1^)^a^	0.01	–
*k*_a(s)_′ (s^–1^)^a^	1	–
*K*_a(s)_ ^a^	0.01	–
*k*_AB(c)_ (M^–1^ s^–1^)^c^	–	90
*k*_AB(s)_ (M^–1^ s^–1^)^c^	67	–
*k*_SrtA_ (s^–1^)^a^	≥10	≥10
*k*_Side_ (s^–1^)^a^	0.12 *k*_SrtA_	0.12 *k*_SrtA_
*K*_d_ (M)^a^	0.0015	0.0015
*k*_d_ (s^–1^)^a^	0.4	0.4
*k*_d_′ (M^–1^s^–1^)^a^	270	270

a Obtained via microkinetic model fitting.

b Measured by ITC.

c Determined from kinetic experiments.

Since the SpyTag-SpyCatcher reaction is the rate-determining step, we first looked into its influence on the catalytic performance as revealed by the initial rate enhancement (*v*_0(c)_/*v*_0(s)_) and the yield gain (Yield_(c)_/Yield_(s)_) in 8 h. Figures 2d–e shows the profile of the initial rates and the yield in 8 h with varying *k*_AB_ for both cascades. With increasing *k*_AB_, both initial rates increase, but the initial rate enhancement decreases from 8 to 4. Similarly, the yields in 8 h increase with an increasing *k*_AB_, but the yield gain decreases from 8 to 1. Not surprisingly, when the reaction is too slow (less than 1 M^–1^s ^–1^), despite a faster rate for catalyzed cascade, the overall rate is so small that the yield remains poor in 8 h. When the reaction is fast enough (∼10^5^ M^–1^ s^–1^), both cascades are accelerated such that spontaneous cascade catches up with catalyzed cascade in 8 h. This trend highlights that the catalytic effect is only observable when the rate-limiting step proceeds at a moderate pace, allowing kinetic differentiation between catalyzed and uncatalyzed pathways. If the reaction is too slow, the system is ineffective regardless of catalysis; if too fast, the catalyst provides little advantage.

If we define a considerable catalytic performance as yield in 8 h > 50%, and *v*_0(c)_/*v*_0(s)_ > 5, we can identify the corresponding window of *k*_AB_ (green region) between 100 and 1000 M^–1^ s^–1^, which suggests that the catalysis is meaningful only when the rate-determining steps proceed at a moderate rate. By contrast, the influence of *k*_SrtA_ is not as significant (Figure 2f and Figure S12). Considerable catalytic performance appears in a broad region of *k*_SrtA_ above 0.2 s^–1^, beyond which the performance plateaus, indicating that this step is not rate-limiting and does not require excessive acceleration. This also supports the use of the engineered SrtA-5 M mutant as a rational and effective choice.

The next important parameters are *K*_a(s)_, *K*_a(c)_, and *K*_d_. The *K*_a(s)_ and *K*_a(c)_ shall ideally follow the relationship as *K*_a(c)_ = *K*_CaM_ *K*_a(s)_, where *K*_CaM_ is the binding constant of CaM to peptide M13. A high *K*_CaM_ implies a pronounced difference between *K*_a(c)_ and *K*_a(s)_ and a profound “gate-opening” event to kick-off the cascade. At a fixed value of *K*_CaM_ at 4.8 × 10^7^ M^–1^, we compare the influence of *K*_a(s)_ on both cascades (Figure 2g and Figure S13). With increasing *K*_a(s)_, the propensity of spontaneous gate-opening increases and the initial rate of the spontaneous cascade exhibits a progressive upward trajectory and crossover with that of catalyzed cascade at a value of ∼0.1, which coincides with the stoichiometry of the catalyst. The crossover is because the catalytic rate is limited by the amount of catalyst in microkinetic modeling. Similar to that of *k*_AB_, we can identify a window of considerable catalytic performance for *K*_a(s)_ between 0.002 and 0.03. Catalysis is meaningful only when both the gating and the gate-opening of SpyTag­(DA) are effective, with the former favoring small *K*_a(s)_ values, and the latter favoring large *K*_a(s)_ values. The simulated profile of *K*_a(c)_-dependent kinetics (Figure 2h) is in excellent agreement with both experimental data and the calculated results based on the rate-determining approximation (Figure S14). Notably, variation in *K*_d_ has little effect on the spontaneous cascade as both [P_s_] and [P] are counted as the final product, but has an important role in the catalyzed cascade by controlling the dissociation of product from the catalyst complex, which directly determines the rate of catalyst regeneration (Figure 2i). At low *K*_d_ values (<10^–6^ M), despite the higher initial rate, the yield in 8 h is limited to ∼10% as the catalyst binds tightly with the product and the reaction halts. The catalysis is meaningful only at sufficiently high *K*_d_ values (>10^–2^ M), and the catalytic performance is plateaued at even higher *K*_d_ values. This plateau is also observed in the dependence of catalytic performance on *k*_SrtA_ and *K*_a(c)_, which suggests that optimization of a catalyzed assembly reaction cascade does not require excessively large values of these parameters. The critical thresholds are often moderate values. Intriguingly, most biological cascades also rely on molecular interactions with moderate binding constants (e.g., 10^6^ M^–1^ for most molecular chaperones in nature toward their substrates,^61–71^ with this affinity range being maintained across different pH and buffer conditions), which is probably essential to maintain a tunable dynamic cascade. Interesting, when *k*_AB_ *T* or *K*_a(c)_[A]_0_ is used as the horizontal axes, the simulated curves can collapse onto composite curves (Figure 2j–l). This shows that there is a synergistic effect between *k*_AB_ and *T*, as well as between *K*_a(c)_ and [A]_0_, in regulating catalytic efficiency (see Supporting Information section Additional Discussions Part C for details). We also looked into proper combination of parameters for optimial catalytic performance whose details are also included in Supporting Information, section Additional Discussions Part D.

### Synergy between Different Catalysts

In reality, tuning these parameters, especially *K*_a(c)_, *k*_SrtA_, and *K*_d_, is sometimes experimentally challenging if not at all impossible. Instead of a fusion protein as the catalyst (e.g., CaM-SrtA), we may also employ a team of individual catalysts (e.g., discrete CaM and SrtA) to fully decouple the activity and concentration for synergy (Figure 3a). This idea is not uncommon in nature where chaperon-like proteins exist in various concentrations and perform distinct jobs such as transporters, assemblers, or folders.^23,32,72,73^ While the spatial proximity in CaM-SrtA could certainly promote the reaction, a team of discrete CaM and SrtA (i.e., a simple physical mixture) enjoys more flexibility in the freely tunable concentrations of CaM or SrtA and thus a broadly accessible scope of thermodynamic driving force for the key steps.

**Figure 3:**
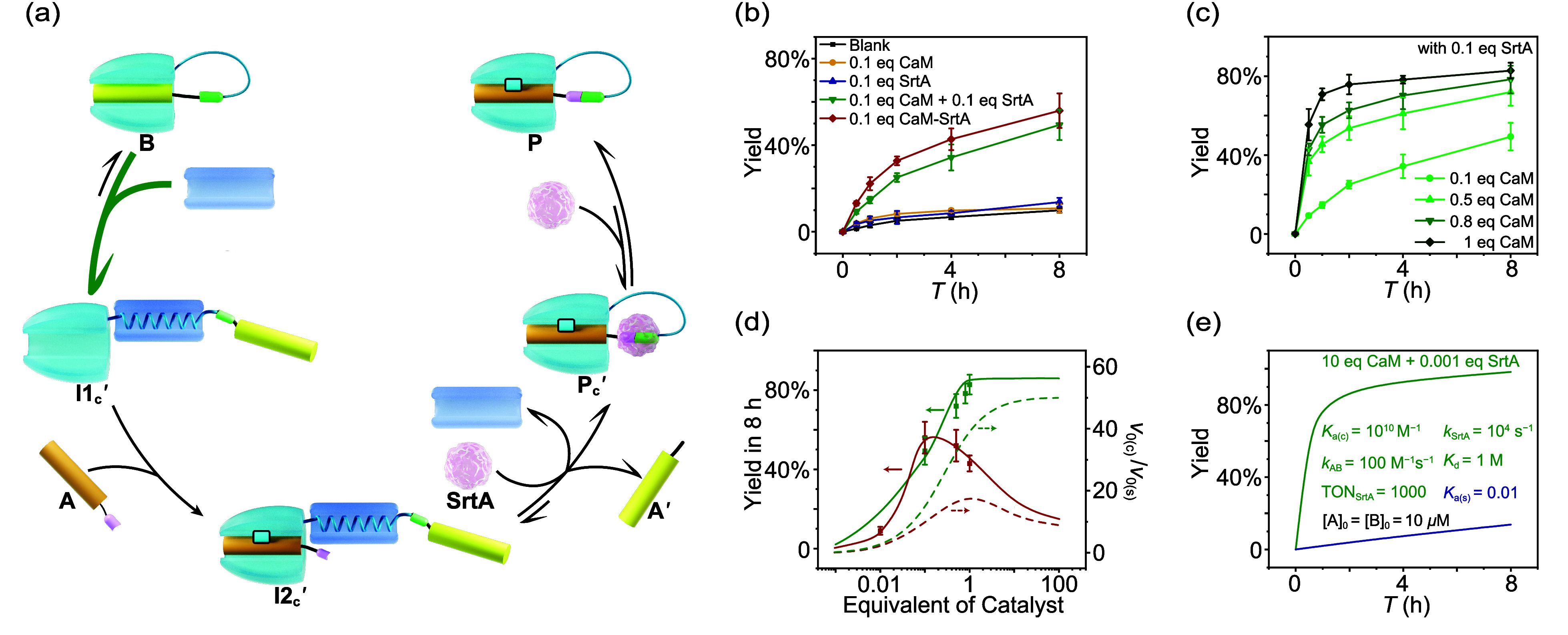
Catalyzed cascade with a physical mixture of CaM and SrtA as a team of catalyst. (a) Schematic illustration of the catalyzed cascade in the presence of a physical mixture of CaM and SrtA. Kinetic profiles of (b) yield *versus T* for a series of control experiments. Substrates **A** and **B** were mixed at 10 μM each in the presence of 0.1 equiv of CaM, or SrtA, or a mixture of CaM and SrtA, or CaM-SrtA, or nothing as a blank control; and (c) yield *versus T* at different equivalents of CaM and a fixed 0.1 equiv of SrtA. (d) Simulated profiles of yield in 8 h (solid line) and *v*_0(c)_/*v*_0(s)_ (dashed line) at different equivalents of catalyst (red: CaM-SrtA; green: varying CaM at a fixed 0.1 equiv of SrtA). Experimental data points are shown as points with an error bar. Arrows point toward the respective vertical axis (left or right) for each data curve. (e) Simulated profiles of an ideal catalyzed cascade (green) and the corresponding spontaneous cascade (blue). Error bars are based on three replicates.

To examine the contribution of each individual domain and reveal the roles of conformational gating and enzymatic ligation separately, we designed a set of nonfused control catalysts, including CaM alone, SrtA alone, and their physical mixture (Figure 3b and Figure S15). When neither CaM nor SrtA was added, **B** struggled to open the gating of SpyTag­(DA) and react with **A**, resulting in a very low yield of less than 10% after 8 h. This sets the baseline for the system without catalytic assistance. Addition of either CaM or SrtA alone did not considerably promote the yield. Specifically, although CaM alone is effective in opening up the gating, it remains tightly bound to the product, preventing the recycling of the catalyst and thus capping the yield at ∼10%. On the other hand, SrtA binds weakly to the unprimed substrates (with a reported *K*_d_ ∼ 10^–3^ M),^74,75^ which is consistent with the mM-range *K*_d_ estimated from our kinetic model fitting, further supporting that SrtA alone can hardly promote the reaction without prior gating. These results suggest that neither gating nor ligation alone is sufficient for efficient catalysis and that both are necessary for a productive cascade.

Impressively, even a physical mixture of CaM and SrtA could increase the yield to 49%, which is slightly lower than the yield of 56% for CaM-SrtA. This result shows that proximity-independent cooperation between CaM and SrtA can also generate a productive reaction pathway. The fact that a nonfused system performs comparably well illustrates a key advantage: the ability to independently tune the concentration of each component, as further demonstrated in Figure 3c and Figure S16. By fixing SrtA at 0.1 equiv and increasing CaM from 0.1 to 1 equiv, we observed a marked increase in both reaction rate and yield in 8 h. This tunability allows CaM to serve as a dynamic handle for modulating the thermodynamic driving force, particularly by accelerating the gate-opening process. Meanwhile, there is also no significant increase in side products in SDS-PAGE, which confirms that the side products are mainly from SrtA-related reactions.

On the other hand, the CaM-SrtA fusion retains unique advantages of its own. As discussed earlier, it encodes a self-contained relay of binding, reaction, and release steps, offering a compact design that simplifies catalyst formulation. While the improvement in yield is modest—likely due to SrtA’s high intrinsic activity—the fusion strategy eliminates the need for external stoichiometric optimization and ensures tight coordination between functional domains. Importantly, these two systems are not meant to be compared in terms of efficiency but rather represent two alternative strategies for catalyst design. The physical mixture offers flexibility in adjusting component ratios, which may be advantageous in the case the intrinsic properties of the component alone could not match the desired performance. In contrast, the fusion design is compact and elegant for easy implementation and facile integration into biological systems via genetic manipulation, particularly in cases where fixed stoichiometry and catalytic performance are desired. Therefore, both configurations have their own merits: the fused construct provides an efficient, ready-to-use catalytic unit, whereas the discrete pair offers great flexibility and broad tunability for systems requiring adaptive optimization.

To gain more insight, we also built a microkinetic model for this teamed catalyst system (Figure S17). Similar to the spontaneous cascade, *K*_d_ has little effect on the observed yield (Figure S18). Profiles of initial rate enhancement and the yield in 8 h versus stoichiometry are illustrated in Figure 3d, representing a stark difference from that of CaM-SrtA. The higher [CaM] is, the faster the reaction is. The yield in 8 h plateaus at ∼80%, and the initial rate enhancement reaches a maximum of 50 at ∼10 equiv of CaM. The simulations are consistent with the experimental results (Figure 3d). We deduced a plausible combination of parameters for an optimal catalytic performance: *K*_a(c)_ = 10^10^ M^–1^, *k*_AB_ = 100 M^–1^ s^–1^, *k*_SrtA_ = 10^4^ s^–1^, and *K*_d_ = 1 M, in the presence of 10 equiv of CaM and 0.001 equiv of SrtA (Figure 3e). The total turnover number can reach ∼10^3^ for SrtA in 8 h, and there are much fewer side products (∼0.1%) as compared to that commonly encountered with a fused protein catalyst (12%). The results highlight the advantages of using a team of catalysts in synergy, indicating that concentration of a particular catalyst may be leveraged to enhance the driving force and achieve the desired performance. Coincidently, there are also many functional biomolecules in nature with distinct kinetic characteristics and versatile roles, such as the chaperone heat shock protein HSP70,^15^ whose expression level can be modulated on demand.^23^

### Free Energy Landscape

We propose the free energy landscapes for all of these scenarios (Figure 4). The relatively low *K*_a(s)_ values in the spontaneous cascade indicate that **I1**_**s**_ exists in a state of higher free energy than that of B with a high kinetic barrier. Conversely, in the presence of the catalyst, the free energy of the first intermediate, **I1**_**c**_ or **I1**_**c**_ **′**, becomes lower than that of B. The reduction of activation energy may be promoted by using a CaM-M13 pair of high affinity or by using large amounts of CaM in the catalyst team. This step is strongly related to the initial rate enhancement. Then, comes the SpyTag-SpyCatcher reaction, which is also the rate-determining step with the largest (but similar) kinetic barrier for all cascades. This step is strongly correlated to the final yield. Subsequent steps are likely to have small kinetic barriers constituting the downhill event in all cascades. Among these, the conversion from **I1**_**c**_ to **I2**_**c**_ is the spontaneous and irreversible formation of isopeptide bonds. According to Howarth and co-workers, the hydrophobic environment created in the SpyTag-SpyCatcher complex is likely to determine that the position of equilibrium lies firmly on the side of bond formation.^38^ While the exact equilibrium and Δ*G* for this step have not been experimentally determined, the process is unambiguously thermodynamically favorable (Δ*G* < 0), confirming that the relative energy levels of the product **I2**_**c**_ is lower than that of the reactant **I1**_**c**_. The same final product was obtained, which confirmed the catalytic role of the catalysts. This model system of assembly reaction cascades shall shed light on the general features of the catalyzed assembly reaction cascades and help understand various chaperone-assisted processes in biological systems.

**Figure 4:**
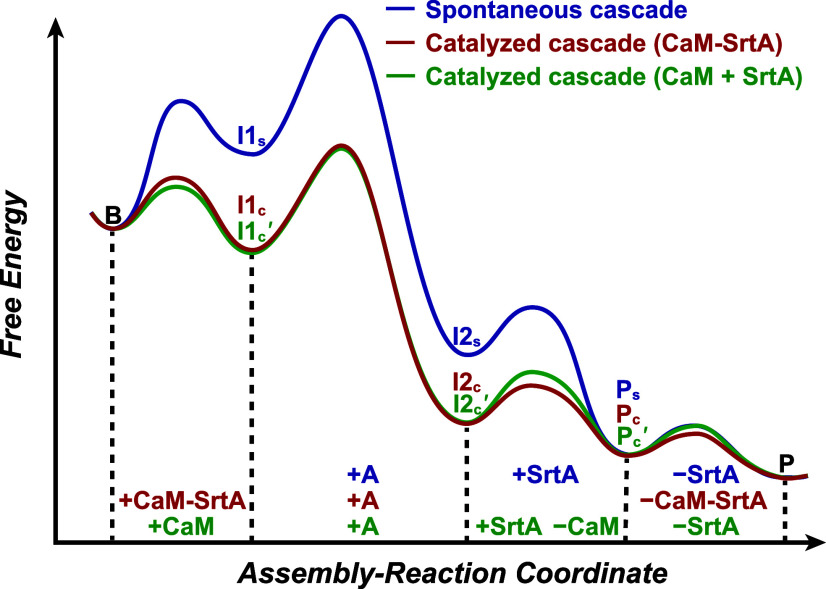
Proposed free energy landscape of the spontaneous and catalyzed cascades. The horizontal axis represents the sequence of steps in the assembly reaction cascades (as shown in Scheme 1f and Figure 3a), and the vertical axis indicates the relative free energy levels, reflecting the spontaneity and energetic favorability of each transformation.

## Conclusion

In summary, we have developed a model assembly reaction cascade that can be accelerated by rationally designed catalysts based on well-established peptide–protein interaction pairs. The cascade combines the noncovalent control of supramolecular assembly as in the conformational regulation of chaperones with the chemical specificity of covalent catalysis as in enzyme-mediated ligations. It is not merely a mimic to either molecular chaperones or enzymes but actually an integration of both within a unified, programmable framework. Notably, spontaneous catalyst regeneration was achieved by SrtA-mediated cyclization with subsequent changes in loop conformation that disfavors the binding of M13 peptide with CaM. Compared to the spontaneous cascade, the catalyzed cascade is ∼12 times faster in terms of the initial rate, and there is ∼17-fold reduction in terms of half-life. Kinetic analysis by microkinetic modeling further reveals the detailed influences of each parameter on the catalytic performance, which suggests ways for optimization by catalyst relay team work. Indeed, the catalyst design as a physical mixture enables flexible tailoring of the concentrations of individual components, offering higher overall catalytic performance at a given set of intrinsic molecular parameters—but often at the cost of increased complexity and higher catalyst loading. By contrast, the catalyst design as a fusion provides a compact, self-contained, genetically encoded solution that functions efficiently at catalytic doses with a built-in mechanism for catalyst regeneration. Both catalyst designs work well, each with its own pros and cons.

As typical hybrid-bonded systems, nature is replete with such catalytic assembly reaction cascades, which mix chemical and physical bonds with a wide range of strengths and dynamics. By reconstruction of a biologically inspired mechanism using defined molecular modules, this work offers a rational approach to emulate and engineer such cascades in synthetic systems. In turn, the insights garnered from these findings offer a valuable framework for understanding natural assembly reaction casacades. In principle, these cascades could be extended to enable programmable signal amplification or regulated protein assembly, and—given its genetic encodability and modularity—it may also be integrated into biological systems and serve as a versatile platform for logic gating or spatiotemporal control in synthetic biology and living systems. From this study, we further draw the following general implications for assembly reaction cascades:

First of all, catalytic performance is superior only within a small window of moderate parameters that are typically encountered in biological systems. *Biology is thus an ideal stage for studying catalyzed assembly reaction cascades*. Natural evolution identifies such a range of kinetic parameters to achieve a highly dynamic and moldable assembly system.^76,77^ As a typical example, most molecular chaperones in nature show binding constants of ∼10^6^ M^–1^, which is neither too low nor too high. Similarly, in DNA-based catalyzed cascades, such as those employing toehold-mediated strand exchange, the precision of kinetics is crucial. Tuning the toehold lengths and their binding affinities is critical for efficient regeneration and sustained turnover of catalysts.^78^ These principles are shared by many artificial systems, where modular design and kinetic tuning drive efficient catalytic performance.^79,80^ Second, an assembly reaction cascade typically spans multiple scales of energy (*k* and *K*), time (*T*), and space (concentration). *The optimal window for catalyzed assembly is thus a collective result of the energy, time, and space of interest*. It is not surprising to see catalysis often work as a team for optimal performance, and their expression level can also be modulated on demand.^81–83^ Third, leveraging conformational changes or catalytic events preceding or coupled with irreversible steps is a key strategy for controlling biological cascades. This concept has been extensively explored, particularly in the context of amyloid and prion formation, where initial conformational transitions or seeding events can trigger irreversible aggregation pathways.^84^ Our work further underscores the potential for rationally designing such interplay in synthetic systems, demonstrating how one can take advantage of the conformational changes and irreversible steps to fulfill a catalytic cycle^23,85^ and how coupling physical assembly with chemical reaction offers a powerful strategy to drive the cascade toward a particular direction.^86^ We believe that this work can inspire the design of new catalysts capable of mediating or accelerating assembly–reaction cascades in both supramolecular and biological systems.

## Supplementary Material



## Notes

All uncropped gel images, ITC results, mass spectra, and full equations used in the microkinetic model are provided. ***Supplementary Methods*** include detailed protocols for DNA construct design, protein expression and purification, protein characterization, and parameter fitting for data set construction in the microkinetic model. All protein sequences are provided in the ***Sequence Information*** section. Figures S1–S18 mentioned in the main text are presented in the ***Supplementary Figures*** section. To facilitate understanding of the ***Kinetic Analysis Using Rate-Determining Approximation*** described in the main text, the related Figures S19–S23, Equations S1–S11, and Table S1 are compiled in this section. Additional discussions—covering (***Part A***) the roles of CaM-SrtA in accelerating the assembly reaction cascade, (***Part B***) the reversibility of SrtA-mediated ligation, (***Part C***) the correlation and synergy between different scales, and (***Part D***) strategies for tuning catalysis toward optimal performance—are organized in the ***Additional Discussions*** section. Figures S24–S27 are included within this section to support the discussions (PDF)
