# Supplementary material for: Designing Catalysts to Accelerate a Protein–Peptide Assembly-Reaction Cascade
Source: ACS Cent Sci. 2025 Jun 16;11(7):1166–77. doi: 10.1021/acscentsci.5c00481 (PMC12291137; doi:10.1021/acscentsci.5c00481)
Supplement: Supplementary file 1 [file oc5c00481_si_001.pdf]

Supporting Information for

Designing Catalysts to Accelerate a Protein-Peptide Assembly-Reaction Cascade

Yibin Sun[a,b], Yue Fang[c], Yajie Liu[a,b], Fengyi Jiang[a,b], Zhichao Lei[a,b], Hanyu Gao[c*], and Wen-Bin Zhang[a,b,d*]

[a] *Department of Polymer Science and Engineering, College of Chemistry and Molecular Engineering, Peking University, Beijing 100871, P. R. China.*

[b] *Beijing National Laboratory for Molecular Sciences, Key Laboratory of Polymer Chemistry & Physics of Ministry of Education, Center for Soft Matter Science and Engineering, Peking University, Beijing 100871, P. R. China.*

[c] *Department of Chemical and Biological Engineering, Hong Kong University of Science and Technology, Clear Water Bay, Hong Kong SAR, P. R. China.*

[d] *Artificial Intelligence for Science-Preferred Program, Shenzhen Graduate School, Peking University, Shenzhen 518055, P. R. China.*

[*] *e-mail: wenbin@pku.edu.cn hanyugao@ust.hk*

Contents

1 Supplementary Methods **S2**

2 Supplementary Figures (Figures S1–S18) **S4**

3 Sequence Information **S18**

4 Kinetic Analysis Using Rate-Determining Approximation (Figures S19–S23, Equations S1–S11, and Table S1) **S21**

5 Additional Discussions (Figures S24–S27) **S29**

1 Supplementary Methods

DNA construction

All oligonucleotide primers were ordered from Genewiz Inc, SZ, and their sequences are provided in **Sequence Information**. Sequences encoding SpyCatcher, YFP, CaM and SrtA were amplified by PCR from the corresponding plasmids previously constructed in our lab. GGG-SpyTag-YFP was cloned into the bacterial co-expression vectors pMCSG19/pRK1037, and other genes were cloned into pET-15b or pQE-80l vectors by standard restriction digestion and ligation protocols. All DNA sequences were confirmed by direct sequencing.

Protein expression and purification

For in situ protease cleavage, the plasmids based on the pMCSG19 vector were introduced into the *E. coli* BL21(DE3) competent cells containing the pRK1037 vector. The single colony was cultured on 2xYT plates containing 100 μg/mL ampicillin and 50 μg/mL kanamycin, and then inoculated into 5 mL of 2xYT broth with the same antibiotics and grown in a shaker (37 °C, 250 rpm). The overnight cultures were inoculated to 250 mL of fresh 2xYT broth containing 100 μg/mL ampicillin and 50 μg/mL kanamycin. When OD_{600} reached 0.8∼1.3, isopropyl-β-D thiogalactopyranoside (IPTG) was added to a concentration of 0.5 mM to induce target protein expression. Then cultures were shaken at 30 °C for 12 h. The other plasmids containing the designed sequences were transformed into BL21(DE3) for expression. Cells were harvested by centrifugation (5000 g $\times$ 30 min, 4 °C).The harvested cell pellets were resuspended in 20-30 mL lysis buffer (50 mM NaH_2PO_4, 300 mM NaCl, 10 mM imidazole, pH 8.0), and lysed by ultrasonication. The supernatant was then collected after centrifugation (10000 g $\times$ 50 min, 4 °C), and mixed with Ni-NTA resin (GE Healthcare. Inc.). The mixture was incubated at 4 °C for 1 h, then loaded to an empty column and washed by wash buffer (50 mM NaH_2PO_4, 300 mM NaCl, 20 mM imidazole, pH 8.0) for five resin volumes, finally eluted by elution buffer (50 mM NaH_2PO_4, 300 mM NaCl, 250 mM imidazole, pH 8.0). The eluted products were collected and further purified by size exclusion chromatography (SEC), which was performed on a Superdex 200 increase 10/300 GL column in an ÄKTA FPLC system (GE Healthcare, Inc.) using TN buffer (150 mM NaCl, 20 mM Tris-HCl, pH 8.0) as the mobile phase at a flow rate of 0.5 mL/min. Protein concentrations were determined by UV absorbance with NanoPhotometer P330 (Implen, Inc.).

Protein characterization

Sodium dodecyl sulfate-polyacrylamide gel electrophoresis (SDS-PAGE) was performed to analyze the protein samples after being mixed with 5 $\times$ SDS-PAGE loading buffer (250 mM

Tris-HCl, 50% glycerol, 10% SDS, 250 mM β-mercaptoethanol, 0.05% bromophenol blue) and heated at 98 °C for 10 min. All SDS-PAGE gels shown in this study were stained with Coomassie Brilliant Blue R-250. Relative protein quantification was performed using Image Lab Software (Bio-Rad) for SDS-PAGE images.

Molecular weight of all samples was confirmed by ultra-performance liquid chromatography-electrospray ionization mass spectrometry (LC-MS) (Waters Corp.) using an Agilent PLRP-S column (300 A, 5 μm, 50 $\times$ 2.1 mm). The separation was performed with a linear gradient from 10% to 90% acetonitrile (containing 0.1% formic acid) in water (containing 0.1% formic acid) over 9 min at a flow rate of 0.2 mL/min. Detection was performed on a quadrupole rods SQ Detector 2 mass spectrometer (Waters Corp.). Data were processed using MassLynx V 4.1 (Waters Corp., Milford, Massachusetts, USA), and the m/z spectrum was converted to the mass spectrum via the MaxEnt1 algorithm (Waters Corp.).

Isothermal titration calorimetry (ITC) experiments were carried out on a MicroCal PEAQ-ITC using a standard 13 injections method. All ITC data were collect at 310 K, 25 mM Tris-HCl, 500 mM NaCl, 10 mM $CaCl_2$, pH 8.0. In order to avoid bias or potentially arbitrary offsets caused by manual adjustment of baseline, all raw data (thermograms) of ITC were integrated by NITPIC (v.1.2.0), fitted in Sedphat (v.12.1b), and visualized through GUSSI (v.1.1.0)[1].

Parameter fitting for dataset construction in microkinetic model

For catalyzed cascade, we initially scrutinized the parameters for the side reaction k_{side}. The parameter k_{side} was permitted to fluctuate across a broad spectrum, from 0.0001 to 1 times of k_{SrtA}, prior to further optimization within a more constricted range of 0.01 to 0.2 times of k_{SrtA}. Sequentially, we delved into the parameters for the inverse reactions, $k_{\mathrm{a(c)}}$ and k_{d}'. Initially, these parameters were allowed to span an expansive range from 1 to 1000000 M^{-1} s^{-1}, followed by a confinement to a more appropriate narrower range. Finally, we generated a set of 100 data points for each parameter within a range from 100 to 10000 M^{-1} s^{-1}, resulting in a comprehensive dataset encompassing 300 data points. We adopted a congruent approach for the spontaneous cascade, wherein three parameters, k_{d}, $K_{\mathrm{a(s)}}$, and $k_{\mathrm{a(s)}}$, were examined in a similar fashion.

2 Supplementary Figures (Figures S1–S18)

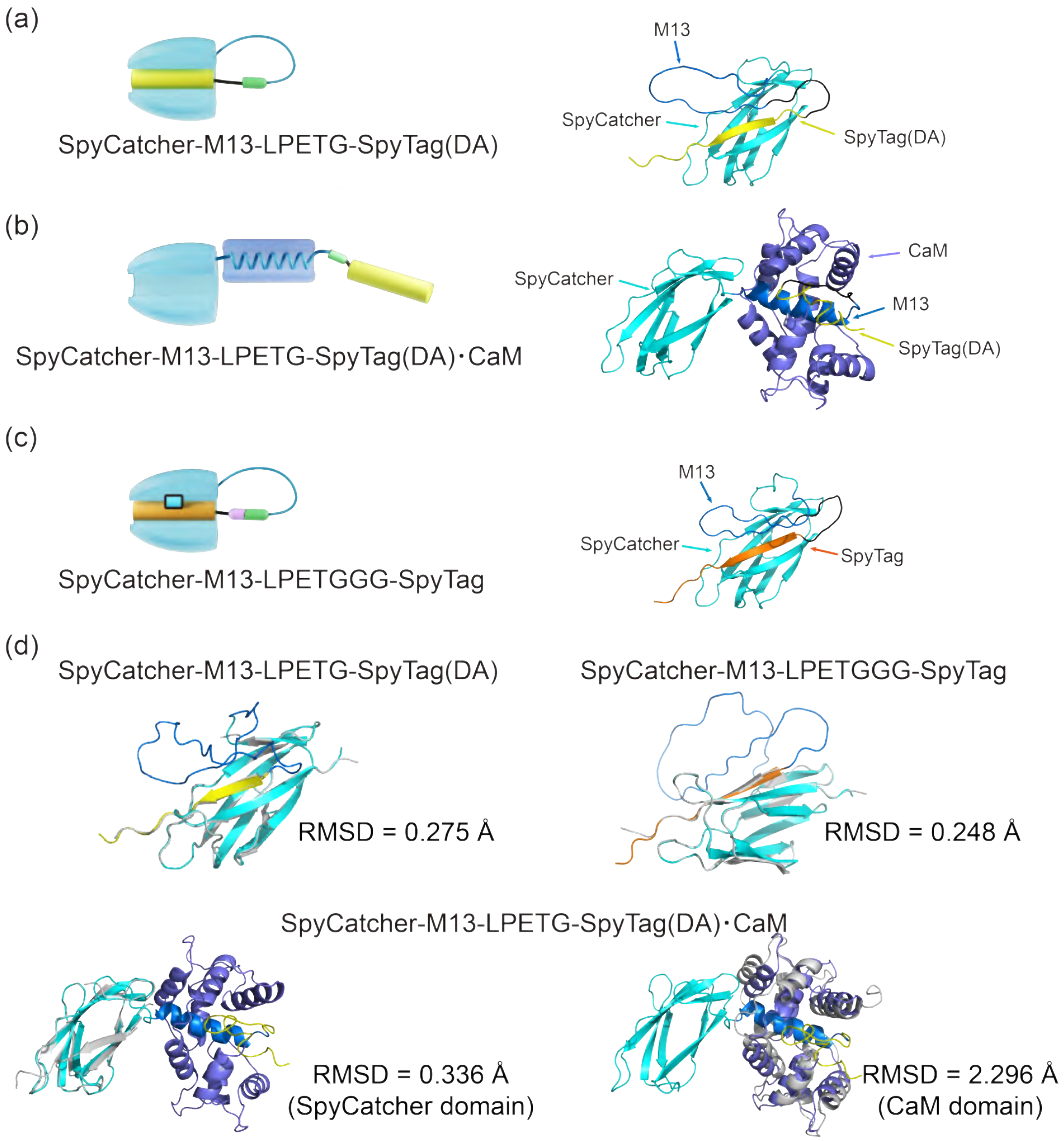

Figure S1: Cartoon illustrations (left) and AlphaFold2 predicted structures[2] (right). **(a)** SpyCatcher-M13-LPETG-SpyTag(DA). **(b)** SpyCatcher-M13-LPETG-SpyTag(DA)•CaM. **(c)** SpyCatcher-M13-LPETGGG-SpyTag. **(d)** Structural alignment of the AF2-predicted model with the corresponding crystal structure domains using PyMOL. The root mean square deviation (RMSD) values were calculated based on Cα atoms. For the SpyCatcher-M13-LPETG-SpyTag(DA)•CaM complex, structural alignment was performed separately for the Spy domain and the CaM domain. It can be found that M13 cannot maintain a α-helix conformation in Figure S1c, therefore CaM domain will auto-detach from the product once the product is formed.

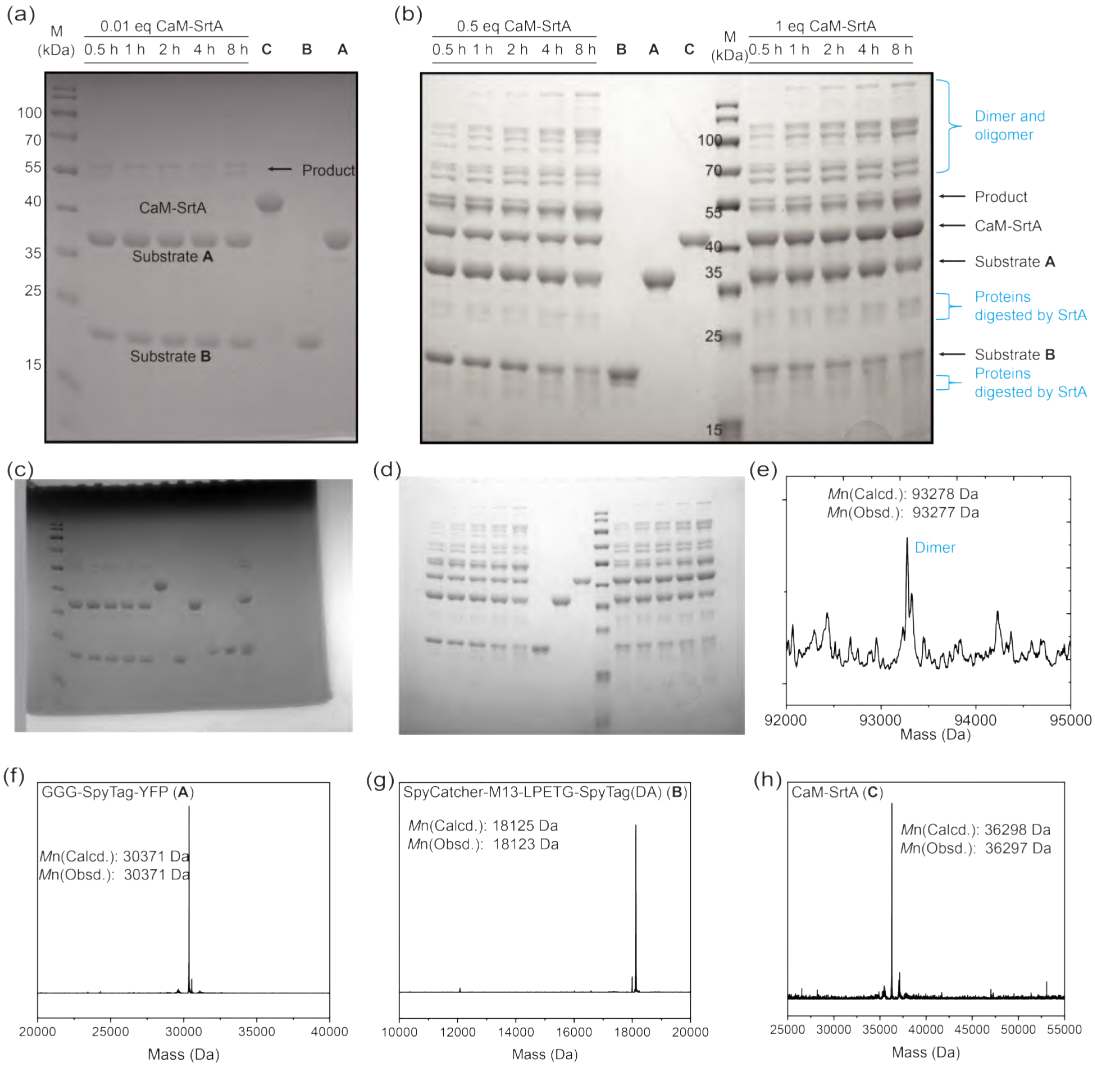

Figure S2: Equivalent-dependent study on CaM-SrtA. **(a)** Investigating the kinetics that **A** and **B** are mixed in 1:1 ratio at 10 μM with 0.01 equivalent of CaM-SrtA by SDS-PAGE (cropped gel). **(b)** Investigating the kinetics that **A** and **B** are mixed in 1:1 ratio at 10 μM with 0.5 (left) or 1 (right) equivalent of CaM-SrtA by SDS-PAGE (cropped gel). When 0.01 equivalent CaM-SrtA was added, a slow generation of product was observed. Addition of 0.5 or 1 equivalent of CaM-SrtA resulted a fast generation of product, but also led to the formation of a large amount of side products. **(c)** Uncropped gel for Figure S2a. **(d)** Uncropped gel for Figure S2b (Buffer condition: 25 mM Tris-HCl, 500 mM NaCl, 10 mM $CaCl_2$, pH 8.0). **(e)** MS spectra of dimer. **(f)** MS spectra of GGG-SpyTag-YFP. **(g)** MS spectra of SpyCatcher-M13-LPETG-SpyTag(DA). **(h)** MS spectra of CaM-SrtA.

To investigate the nature of the side products observed in SDS-PAGE, we performed mass spectrometry. However, due to the detection limits of the instrument, species larger than 70 kDa, especially at low abundance, could hardly be detected. Despite repeated attempts, only the dimeric species (70–100 kDa, Figure S2e) were reliably detected. These results support our conclusion that the major side reaction arises from protein oligomerization, as illustrated in Scheme 1g. In our quantification of reaction yield, all peaks in the 70–100 kDa range were grouped and approximated as dimers. Their relative abundance was low, and this approximation had negligible impact on the overall yield calculation. It is also worth noting that dimer and oligomer formation is a well-documented outcome in protein cyclization systems, particularly under conditions involving reactive termini. Similar examples have been reported in the literature[3–5].

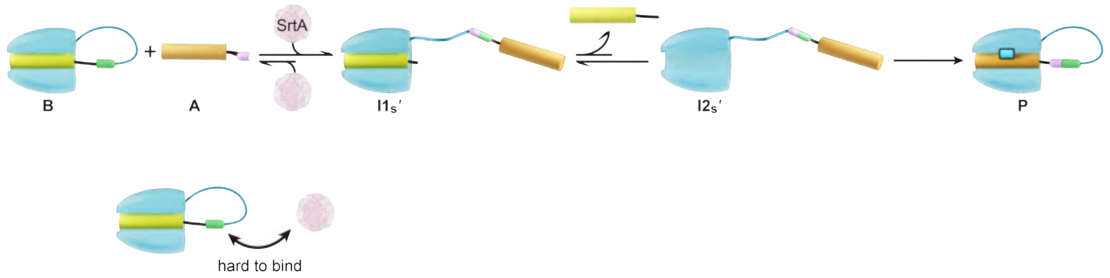

Figure S3: Illustration of the unfavorable pathway of spontaneous cascade. In this pathway, SrtA-mediated ligation proceeds firstly before the dissociation of SpyCatcher•SpyTag(DA) complex, giving $\mathbf{I1'_s}$ complex as intermediate; then SpyTag expels SpyTag(DA) and reacts with SpyCatcher to give **P**. If this is the case, the formation of intermediate $\mathbf{I1'_s}$ could be investigated, as SrtA-mediated ligation is a fast and the dissociation of SpyCatcher•SpyTag(DA) complex is relatively slow, leading to the accumulation of intermediate $\mathbf{I1'_s}$. While a molecular weight of 46657 Da, referring to the formation of $\mathbf{I1'_s}$, is not observed in LC-MS spectrum. Therefore, this also shows the SrtA-mediated ligation in not easy to proceed before the dissociation of SpyCatcher•SpyTag(DA) complex, probably because the LPETG group in substrate **B** is protected by the loop-shape M13, making the substrate hard to be attacked by SrtA directly.

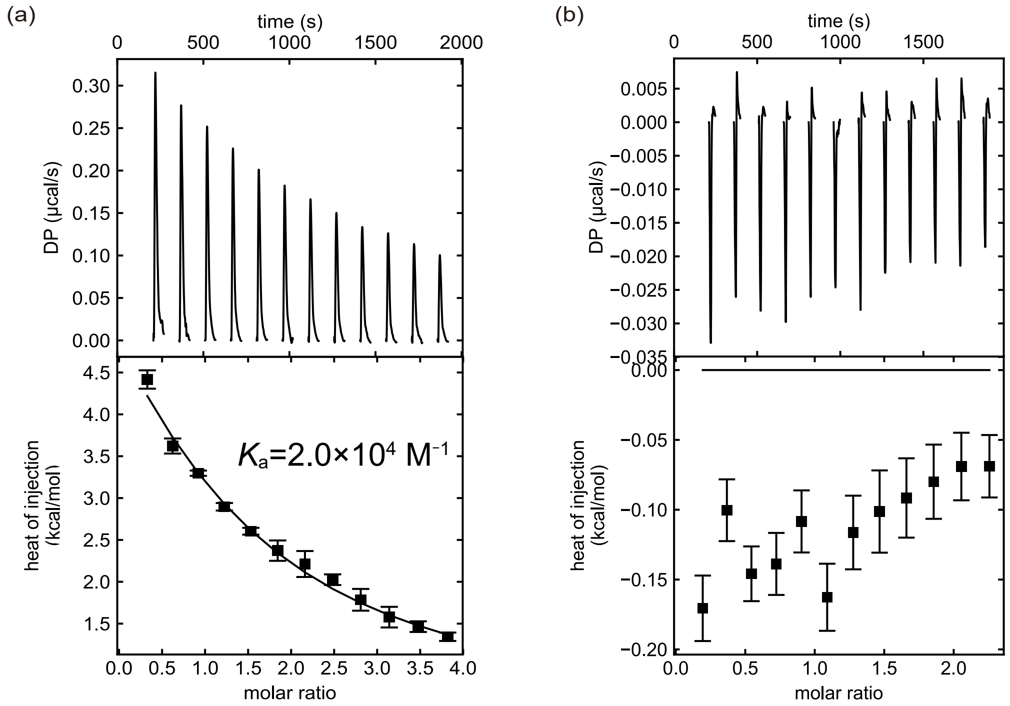

Figure S4: Isothermal titration thermograms. **(a)** SpyCatcher-M13-2-LPETG-SpyTag(DA) (0.4 mM) titrated into CaM (0.02 mM). **(b)** SpyCatcher-M13-3-LPETG-SpyTag(DA) (0.5 mM) titrated into CaM (0.04 mM). (310 K, 25 mM Tris-HCl, 500 mM NaCl, 10 mM $CaCl_2$, pH 8.0)

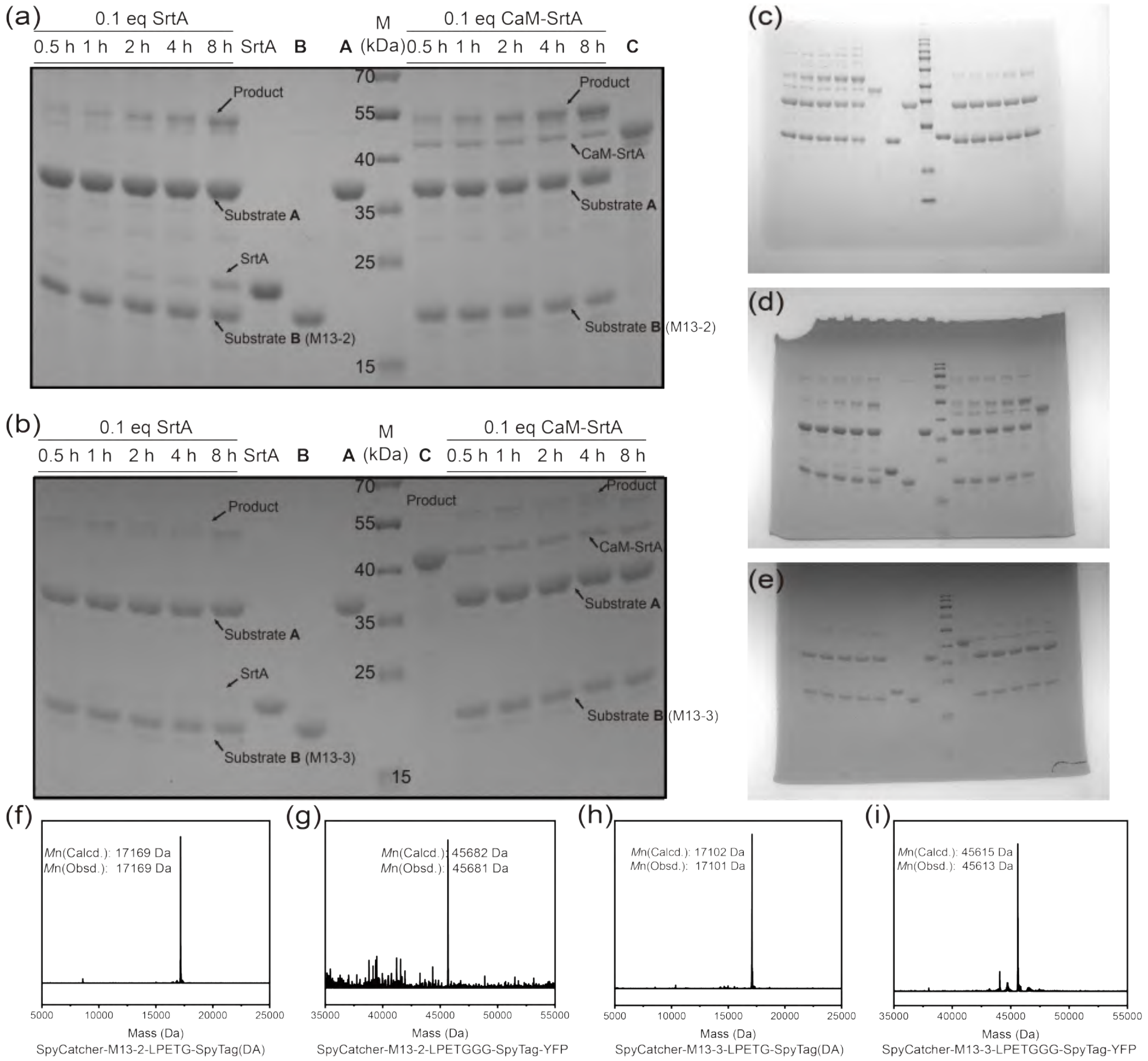

Figure S5: Investigating the kinetics of spontaneous cascade (0.1 eq SrtA) and catalyzed cascade (0.1 eq CaM-SrtA) of M13-dependent experiment by SDS-PAGE. **(a)** SpyCatcher-M13-2-LPETG-SpyTag(DA) serves as substrate (cropped gel). **(b)** SpyCatcher-M13-3-LPETG-SpyTag(DA) serves as substrate (cropped gels). **(c)** Uncropped gel for Figure 1a. **(d)** Uncropped gel for Figure S5a. **(e)** Uncropped gel for Figure S5b. (Buffer condition: 25 mM Tris-HCl, 500 mM NaCl, 10 mM $CaCl_2$, pH 8.0) **(f)** MS spectra of SpyCatcher-M13-2-LPETG-SpyTag(DA). **(g)** MS spectra of SpyCatcher-M13-2-LPETGGG-SpyTag-YFP. **(h)** MS spectra of SpyCatcher-M13-3-LPETG-SpyTag(DA). **(i)** MS spectra of SpyCatcher-M13-3-LPETGGG-SpyTag-YFP.

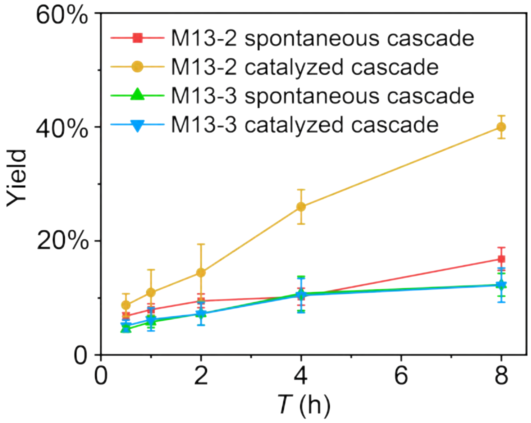

Figure S6: Kinetic profiles of yield *versus* time (T) extracted from Figure S5. Error bars show range from 3 repeated experiments.

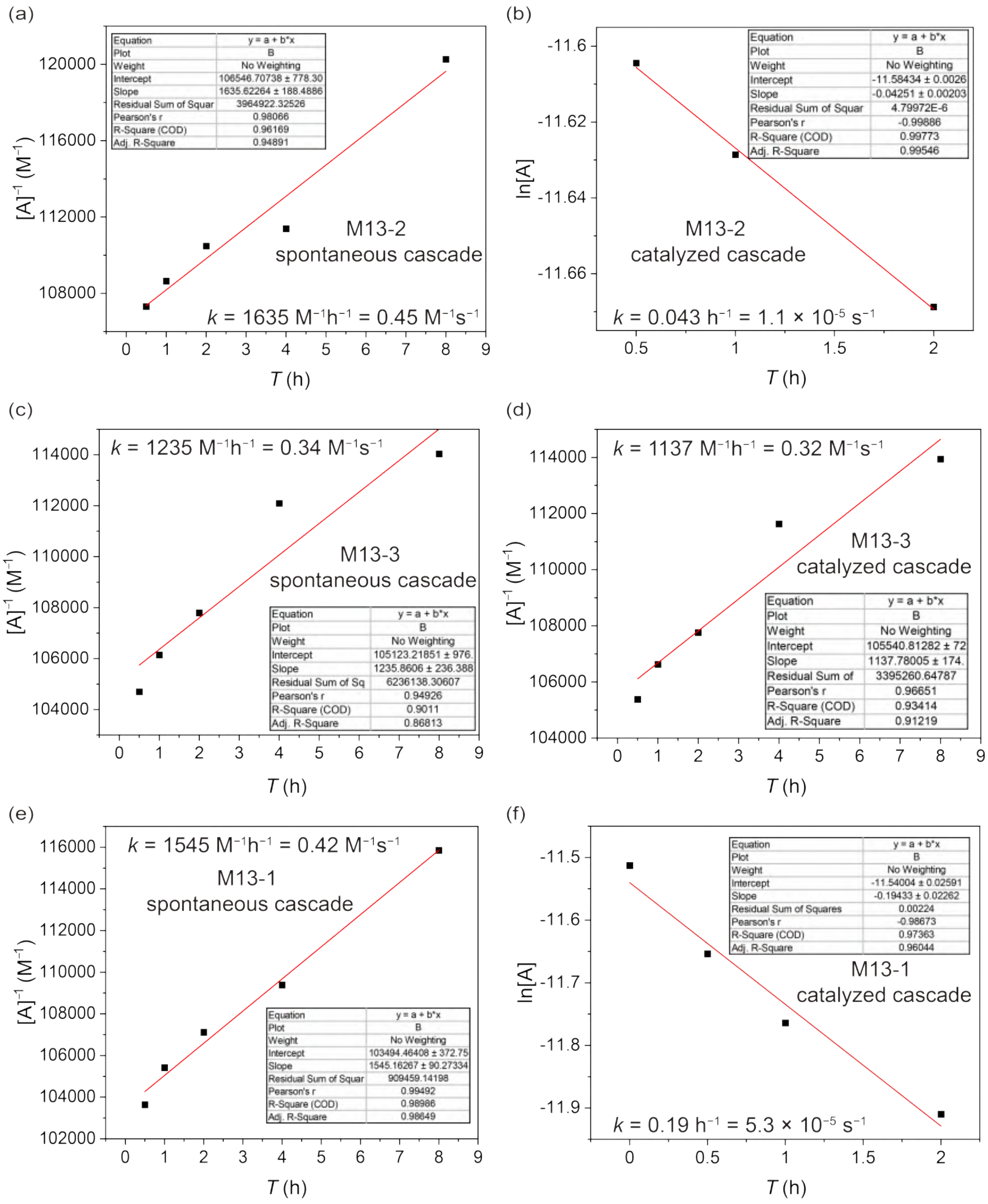

Figure S7: Linear fitting of the kinetic data extracted from Figure S6 and Figure 1b using second order kinetic model for spontaneous cascade and first order kinetic model for catalyzed cascade.

$$\frac{d[\mathrm{B}]}{dt} = -k_{\mathrm{a(s)}}[\mathrm{B}] + k_{\mathrm{a(s)}}'[\mathrm{I1_s}]$$

$$\frac{d[\mathrm{I1_s}]}{dt} = k_{\mathrm{a(s)}}[\mathrm{B}] - k_{\mathrm{a(s)}}'[\mathrm{I1_s}] - k_{\mathrm{AB\,(s)}}[\mathrm{I1_s}][\mathrm{A}]$$

$$\frac{d[\mathrm{A}]}{dt} = -k_{\mathrm{AB\,(s)}}[\mathrm{I1_s}][\mathrm{A}]$$

$$\frac{d[\mathrm{I2_s}]}{dt} = k_{\mathrm{AB\,(s)}}[\mathrm{I1_s}][\mathrm{A}] - k_{\mathrm{SrtA}}[\mathrm{I2_s}] - 2k_{\mathrm{side}}[\mathrm{I2_s}]$$

$$\frac{d[\mathrm{P_s}]}{dt} = k_{\mathrm{SrtA}}[\mathrm{I2_s}] - k_{\mathrm{d}}[\mathrm{P_s}] + k_{\mathrm{d}}'[\mathrm{P_s}][\mathrm{SrtA}]$$

$$\frac{d[\mathrm{P}]}{dt} = k_{\mathrm{d}}[\mathrm{P_s}] - k_{\mathrm{d}}'[\mathrm{P_s}][\mathrm{SrtA}]$$

$$\frac{d[\mathrm{A}']}{dt} = k_{\mathrm{SrtA}}[\mathrm{I2_s}] + k_{\mathrm{side}}[\mathrm{I2_s}]$$

$$\frac{d[\mathrm{P_{side}}]}{dt} = k_{\mathrm{side}}[\mathrm{I2_s}]$$

Figure S8: Reaction rates of spontaneous cascade via microkinetic modeling.

$$\frac{d[\mathrm{B}]}{dt} = -k_{\mathrm{a(c)}}[\mathrm{B}][\mathrm{C}] + k_{\mathrm{a(c)}}'[\mathrm{I1_c}]$$

$$\frac{d[\mathrm{C}]}{dt} = -k_{\mathrm{a(c)}}[\mathrm{B}][\mathrm{C}] + k_{\mathrm{a(c)}}'[\mathrm{I1_c}] + k_{\mathrm{d}}[\mathrm{P_c}] - k_{\mathrm{d}}'[\mathrm{P}][\mathrm{C}]$$

$$\frac{d[\mathrm{I1_c}]}{dt} = k_{\mathrm{a(c)}}[\mathrm{B}][\mathrm{C}] - k_{\mathrm{a(c)}}'[\mathrm{I1_c}] - k_{\mathrm{AB\,(c)}}[\mathrm{I1_c}][\mathrm{A}]$$

$$\frac{d[\mathrm{A}]}{dt} = -k_{\mathrm{AB\,(c)}}[\mathrm{I1_c}][\mathrm{A}]$$

$$\frac{d[\mathrm{I2_c}]}{dt} = k_{\mathrm{AB\,(c)}}[\mathrm{I1_c}][\mathrm{A}] - k_{\mathrm{SrtA}}[\mathrm{I2_c}] - 2k_{\mathrm{side}}[\mathrm{I2_c}]$$

$$\frac{d[\mathrm{P_c}]}{dt} = k_{\mathrm{SrtA}}[\mathrm{I2_c}] - k_{\mathrm{d}}[\mathrm{P_c}] + k_{\mathrm{d}}'[\mathrm{P}][\mathrm{C}]$$

$$\frac{d[\mathrm{A}']}{dt} = k_{\mathrm{SrtA}}[\mathrm{I2_c}] + k_{\mathrm{side}}[\mathrm{I2_c}]$$

$$\frac{d[\mathrm{P}]}{dt} = k_{\mathrm{d}}[\mathrm{P_c}] - k_{\mathrm{d}}'[\mathrm{P}][\mathrm{C}]$$

$$\frac{d[\mathrm{P_{side}}]}{dt} = k_{\mathrm{side}}[\mathrm{I2_c}]$$

Figure S9: Reaction rates of catalyzed cascade via microkinetic modeling.

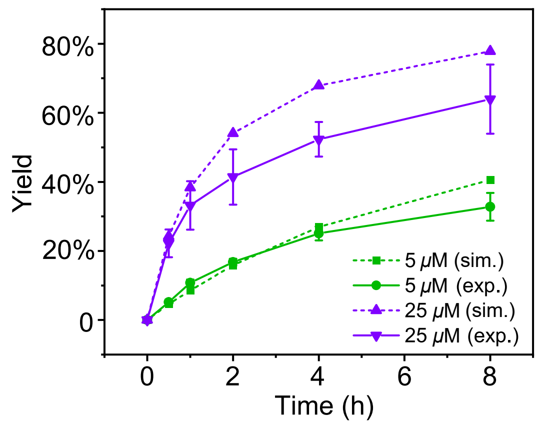

Figure S10: Simulated (dashed line) and experimentally obtained (solid line) kinetic profiles of spontaneous and catalyzed cascades with initial substrate concentrations of 5 μM (green) and 25 μM (purple). Error bars show range from 3 repeated experiments.

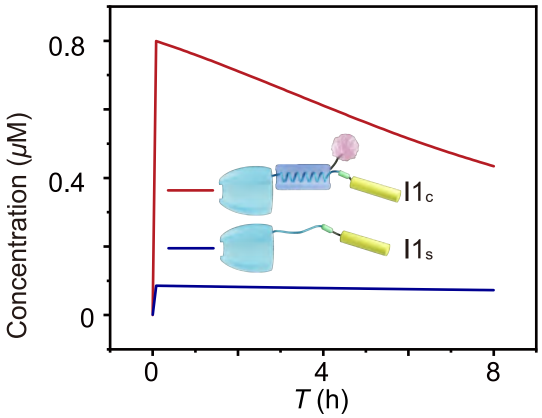

Figure S11: Simulated concentration *versus* time (T) of the intermediates $\mathbf{I1}_s$ in spontaneous cascade (blue) and $\mathbf{I1}_c$ in catalyzed cascade (red). (0.1 equivalent of CaM-SrtA, $[A]_0 = [B]_0 = 10$ μ M). The "gate-opening" intermediate $\mathbf{I1}_c$ reaches a maximum concentration of 0.8 μ M shortly after catalyzed cascade onset, followed by a gradual decrease; whereas $\mathbf{I1}_s$ in spontaneous cascade peaks at 0.1 μ M. The rise in the concentration of $\mathbf{I1}_c$ in comparison to $\mathbf{I1}_s$ affords a compelling evidence indicating that the catalyst accelerates the kinetics. The acceleration is achieved by elevating the concentration of free SpyCatcher domain, which is capable of covalent binding with substrate **A**, thereby facilitating the progression of the assembly-reaction cascade.

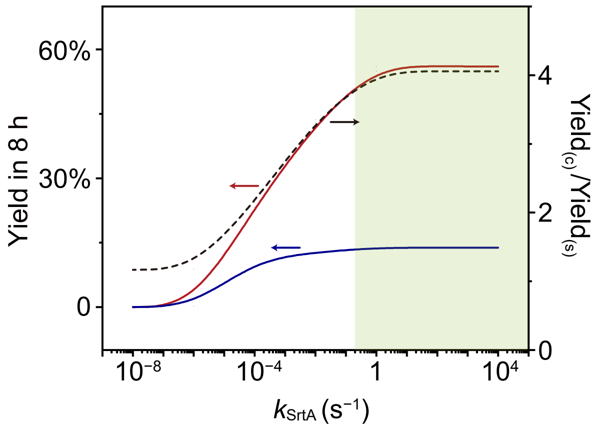

Figure S12: Simulated profiles of yield in 8 h *versus* k_{SrtA} of spontaneous and catalyzed cascades, and the corresponding yield gain ($\mathrm{Yield}_{(c)}/\mathrm{Yield}_{(s)}$) in 8 h (0.1 equivalent of CaM-SrtA, $[A]_0 = [B]_0 = 10\ \mu$ M).

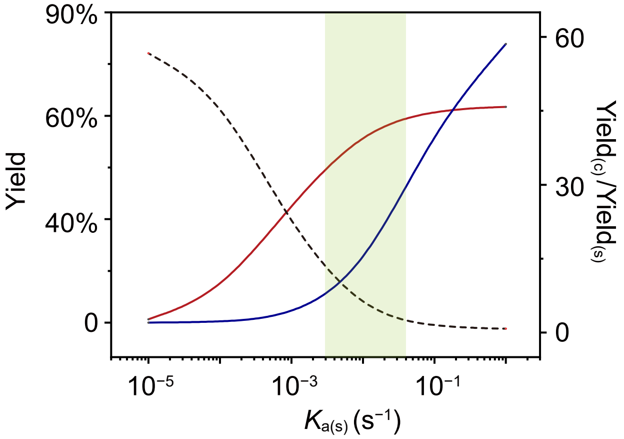

Figure S13: Simulated profiles of yield in 8 h *versus* $K_{\mathrm{a(s)}}$ of spontaneous and catalyzed cascades, and the corresponding yield gain ($\mathrm{Yield}_{(c)}/\mathrm{Yield}_{(s)}$) in 8 h (0.1 equivalent of CaM-SrtA, $[A]_0 = [B]_0 = 10\ \mu$ M).

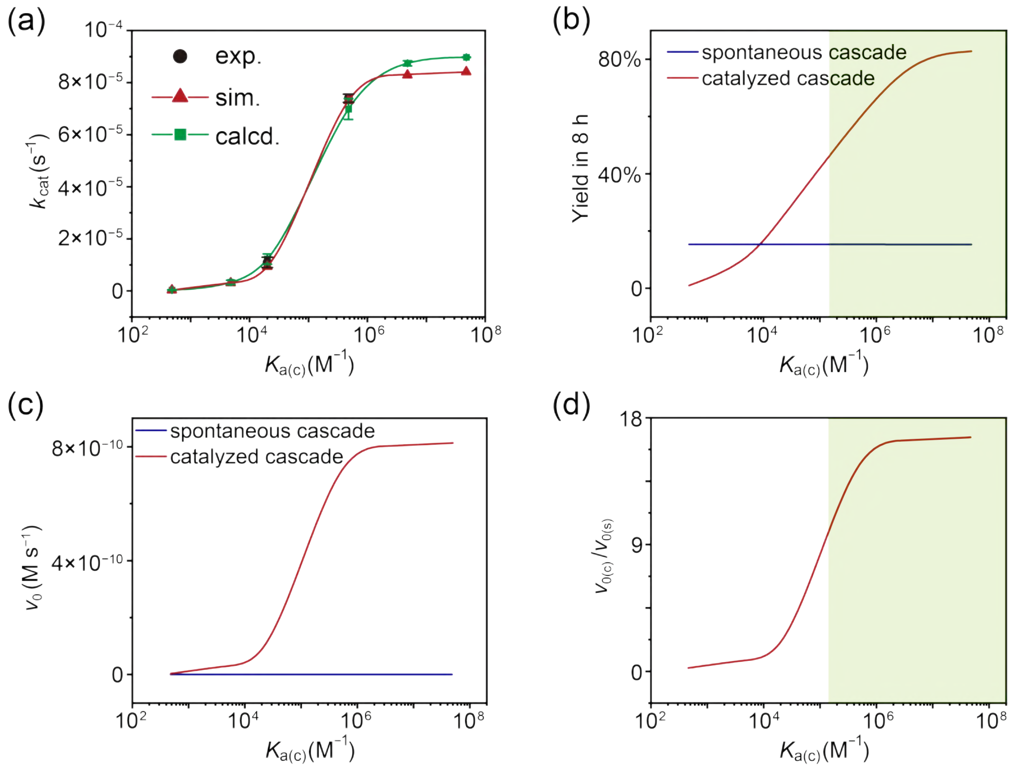

Figure S14: Revealing the rule of $K_{a(c)}$ governing the catalytic performance. **(a)** Experimentally obtained (black), simulated (red), and calculated (green, details can be found in section **Kinetic Analysis Using Rate-Determining Approximation**) profiles of k_{cat} *versus* $K_{a(c)}$ using first-order kinetic model. **(b)** Simulated profile of yield in 8 h *versus* $K_{a(c)}$. **(c)** Simulated profile of v_0 *versus* $K_{a(c)}$. **(d)** Simulated profile of $v_{0(c)}/v_{0(s)}$ *versus* $K_{a(c)}$. (0.1 equivalent of CaM-SrtA, $[A]_0 = [B]_0 = 10\ \mu M$)

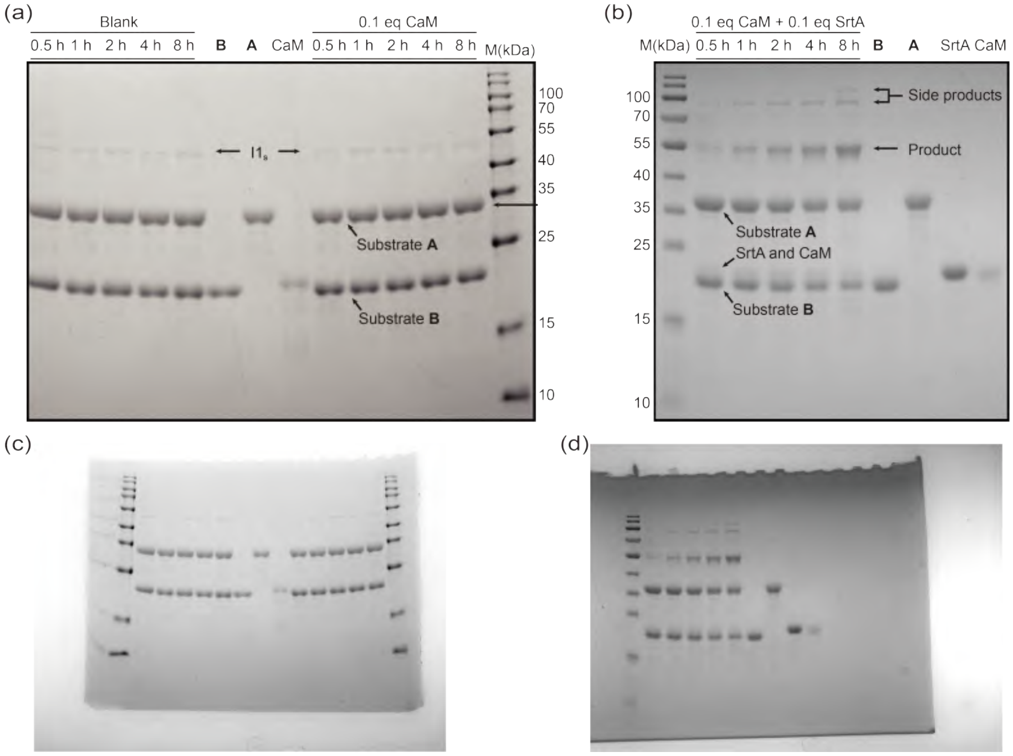

Figure S15: Investigating the kinetics of control experiments. **(a)** **A** and **B** are mixed in 1:1 ratio at 10 μM with (right) or without (left) 0.1 equivalent CaM by SDS-PAGE (cropped gel). **(b)** **A** and **B** are mixed in 1:1 ratio at 10 μM with 0.1 equivalent CaM and 0.1 equivalent SrtA by SDS-PAGE (cropped gel). **(c)** Uncropped gel for Figure S15a. **(d)** Uncropped gel for Figure S15b. (Buffer condition: 25 mM Tris-HCl, 500 mM NaCl, 10 mM $CaCl_2$, pH 8.0)

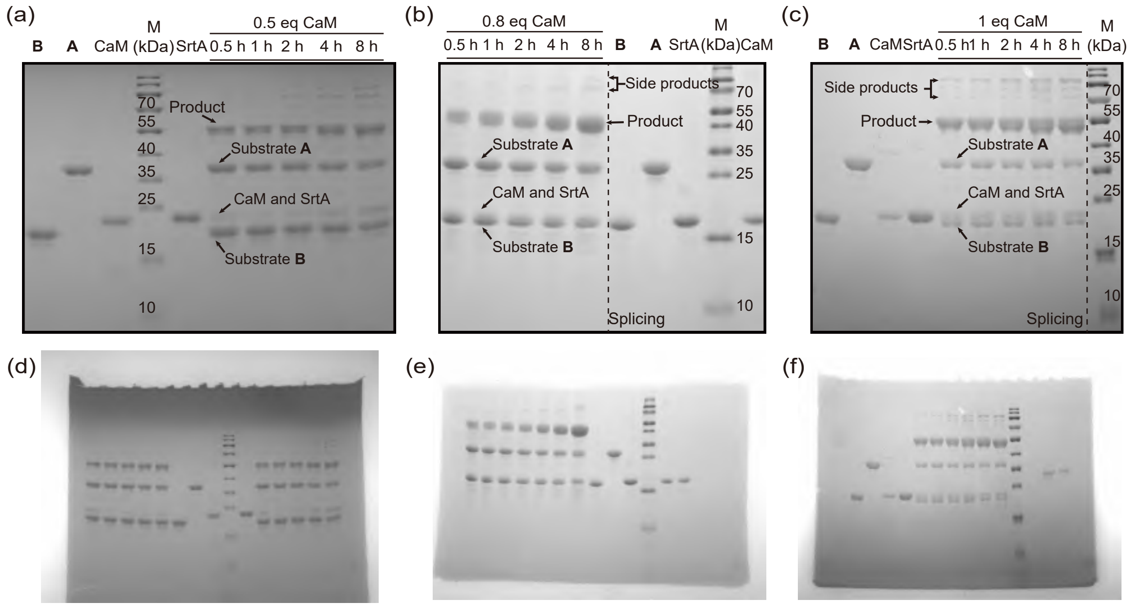

Figure S16: Equivalent-dependent study on CaM by SDS-PAGE. In all experiments, **A** and **B** were mixed in 1:1 ratio at 10 μM in presence of 0.1 equivalent of SrtA. **(a)** 0.5 equivalent of CaM (cropped gel). **(b)** 0.8 equivalent of CaM (spliced gel). **(c)** 1 equivalent of CaM (spliced gel). **(d)** Uncropped gel for Figure S15a. **(e)** Full-size gel for Figure S15b. **(f)** Full-size gel for Figure S15c. (Buffer condition: 25 mM Tris-HCl, 500 mM NaCl, 10 mM $CaCl_2$, pH 8.0)

$$\mathrm{B} + \mathrm{CaM} \underset{k_{\mathrm{a(c)}}'}{\overset{k_{\mathrm{a(c)}}}{\rightleftharpoons}} \mathrm{I1_c}'$$

$$\mathrm{I1_c}' + \mathrm{A} \xrightarrow{k_{\mathrm{AB(c)}}} \mathrm{I2_c}'$$

$$\mathrm{I2_c}' + \mathrm{SrtA} \xrightarrow{k_{\mathrm{SrtA}}} \mathrm{P_c}' + \mathrm{A}' + \mathrm{CaM}$$

$$\mathrm{P_c}' \underset{k_{\mathrm{d}}'}{\overset{k_{\mathrm{d}}}{\rightleftharpoons}} \mathrm{P} + \mathrm{SrtA}$$

$$n\mathrm{I2_c}' \xrightarrow{k_{\mathrm{side}}} \mathrm{P_{side}} + n\mathrm{A}'$$

$$\frac{d[\mathrm{B}]}{dt} = -k_{\mathrm{a(c)}}[\mathrm{B}][\mathrm{CaM}] + k_{\mathrm{a(c)}}'[\mathrm{I1_c}']$$

$$\frac{d[\mathrm{CaM}]}{dt} = -k_{\mathrm{a(c)}}[\mathrm{B}][\mathrm{CaM}] + k_{\mathrm{a(c)}}'[\mathrm{I1_c}'] + k_{\mathrm{SrtA}}[\mathrm{I2_c}']$$

$$\frac{d[\mathrm{I1_c}']}{dt} = k_{\mathrm{a(c)}}[\mathrm{B}][\mathrm{CaM}] - k_{\mathrm{a(c)}}'[\mathrm{I1_c}'] - k_{\mathrm{AB\,(c)}}[\mathrm{I1_c}'][\mathrm{A}]$$

$$\frac{d[\mathrm{A}]}{dt} = -k_{\mathrm{AB\,(c)}}[\mathrm{I1_c}'][\mathrm{A}]$$

$$\frac{d[\mathrm{I2_c}']}{dt} = k_{\mathrm{AB\,(c)}}[\mathrm{I1_c}'][\mathrm{A}] - k_{\mathrm{SrtA}}[\mathrm{I2_c}'] - 2k_{\mathrm{side}}[\mathrm{I2_c}']$$

$$\frac{d[\mathrm{P_c}']}{dt} = k_{\mathrm{SrtA}}[\mathrm{I2_c}'] - k_{\mathrm{d}}[\mathrm{P_c}'] + k_{\mathrm{d}}'[\mathrm{P}][\mathrm{SrtA}]$$

$$\frac{d[\mathrm{A}']}{dt} = k_{\mathrm{SrtA}}[\mathrm{I2_c}'] + k_{\mathrm{side}}[\mathrm{I2_c}']$$

$$\frac{d[\mathrm{P}]}{dt} = k_{\mathrm{d}}[\mathrm{P_c}'] - k_{\mathrm{d}}'[\mathrm{P}'][\mathrm{SrtA}]$$

$$\frac{d[\mathrm{P_{side}}]}{dt} = k_{\mathrm{side}}[\mathrm{I2_c}']$$

Figure S17: The microkinetic model of catalyzed cascade using unfused CaM and SrtA.

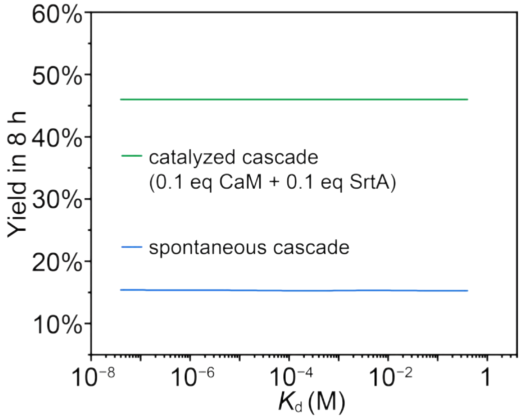

Figure S18: Simulated profile of yield in 8 h *versus* K_{d}.

3 Sequence Information

GGG-SpyTag-YFP

001 MKGSSETVRFQ/GGGVDAHIVMVDAYKPTKSGGSGSELMSK
041 GEELFTGVVPILVELDGDVNGHKFSVSGEGEGDATYGKLT
081 LKLLCTTGKLPVPWPTLVTTLGYGVQCFARYPDHMKQHDF
121 FKSAMPEGYVQERTIFFKDDGNYKTRAEVKFEGDTLVNRI
161 ELKGIDFKEDGNILGHKLEYNYNSHNVYITADKQKNGIKA
201 NFKIRHNIEDGGVQLADHYQQNTPIGDGPVLLPDNHYLSY
241 QSALFKDPNEKRDHMVLLEFLTAAGITEGMNELYKLEHHH
281 HHH

The constructs were cloned into pMCSG19 vectors (encoding MKGSSETVRFQ/GGG-A-YFP proteins) and expressed in E. coli BL21 harboring the pRK1037 plasmid, which co-expresses TVMV protease to cleave the MKGSSETVRFQ tag and yield the N-terminal GGG sequence[6].

SpyCatcher-M13-1-LPETG-SpyTag(DA)

001 MKGSSHHHHHHVEASVTTLSGLSGEQGPSGDMTTEEDSAT
041 HIKFSKRDEDGRELAGATMELRDSSGKTISTWISDGHVKD
081 FYLYPGKYTFVETAAPDGYEVATAITFTVNEQGQVTVNGE
121 ATKGTLRRGQILWNLGLNRIQTQIKLPETGGSVPTIVMVA
161 AYKRYK

In particular, all references to "SpyTag(DA)" in this study specifically denote the "SpyTag002(DA)" variant[1]. This particular variant was selected because: (1) its enhanced binding to SpyCatcher relative to wild-type SpyTag, and (2) its slower displacement kinetics better demonstrate the catalytic advantage in our system.

SpyCatcher-M13-1-LPETGGG-SpyTag-YFP

001 MKGSSHHHHHHVEASVTTLSGLSGEQGPSGDMTTEEDSAT
041 HIKFSKRDEDGRELAGATMELRDSSGKTISTWISDGHVKD
081 FYLYPGKYTFVETAAPDGYEVATAITFTVNEQGQVTVNGE
121 ATKGTLRRGQILWNLGLNRIQTQIKLPETGGGVDAHIVMV
161 DAYKPTKSGGSGSELMSKGEELFTGVVPILVELDGDVNGH
201 KFSVSGEGEGDATYGKLTLKLLCTTGKLPVPWPTLVTTLG

241 YGVQCFARYPDHMKQHDFFKSAMPEGYVQERTIFFKDDGN
281 YKTRAEVKFEGDTLVNRIELKGIDFKEDGNILGHKLEYNY
321 NSHNVYITADKQKNGIKANFKIRHNIEDGGVQLADHYQQN
361 TPIGDGPVLLPDNHYLSYQSALFKDPNEKRDHMVLLEFLT
401 AAGITEGMNELYKLEHHHHHH

SpyCatcher-M13-2-LPETG-SpyTag(DA)

001 MKGSSHHHHHHVEASVTTLSGLSGEQGPSGDMTTEEDSAT
041 HIKFSKRDEDGRELAGATMELRDSSGKTISTWISDGHVKD
081 FYLYPGKYTFVETAAPDGYEVATAITFTVNEQGQVTVNGE
121 ATKGTINLKALAALAQIILLPETGGSVPTIVMVAAYKRYK

SpyCatcher-M13-3-LPETG-SpyTag(DA)

001 MKGSSHHHHHHVEASVTTLSGLSGEQGPSGDMTTEEDSAT
041 HIKFSKRDEDGRELAGATMELRDSSGKTISTWISDGHVKD
081 FYLYPGKYTFVETAAPDGYEVATAITFTVNEQGQVTVNGE
121 ATKGTNLGLNRIQTQIKLPETGGSVPTIVMVAAYKRYK

To investigate how CaM-M13 binding affinity affects catalytic efficiency, we screened a series of M13 variants. Truncating the peptide proved to be a convenient and reliable way to tune binding affinity, enabling systematic comparison while minimizing unrelated variables. M13-2 (shorter sequence) exhibits weaker binding due to reduced hydrophobic interactions, and M13-3 (even shorter and lacking the critical Trp residue) loses CaM-binding capability. The chosen variants provided a stable, well-behaved set of constructs with different CaM affinities, suitable for validating the predictions of our microkinetic model.

Our primary goal here was to examine the kinetic consequence of different binding affinities, not to dissect how specific sequence changes determine affinity. These three variants thus provided a practical and sufficient basis for correlating binding constants with catalytic performance within our microkinetic model.

SrtA

001 MGQAKPQIPKDKSKVAGYIEIPDADIKEPVYPGPATREQL
041 NRGVSFAEENESLDDQNISIAGHTFIDRPNYQFTNLKAAK
081 KGSMVYFKVGNETRKYKMTSIRNVKPTAVEVLDEQKGKDK
121 QLTLITCDDYNEETGVWETRKIFVATEVKHHHHHH

CaM

001 MKGSSHHHHHHVEASADQLTEEQIAEFKEAFSLFDKDGDG
041 TITTKELGTVMRSLGQNPTEAELQDMINEVDADGNGTIDF
081 PEFLTMMARKMKDTDSEEEIREAFRVFDKDGNGYISAAEL
121 RHVMTNLGEKLTDEEVDEMIREADIDGDGQVNYEEFVTMM
161 TSK

CaM-SrtA

001 MKGSSHHHHHHVEASADQLTEEQIAEFKEAFSLFDKDGDG
041 TITTKELGTVMRSLGQNPTEAELQDMINEVDADGNGTIDF
081 PEFLTMMARKMKDTDSEEEIREAFRVFDKDGNGYISAAEL
121 RHVMTNLGEKLTDEEVDEMIREADIDGDGQVNYEEFVTMM
161 TSKGTGGGSGGGSGGGSGGGSGSQAKPQIPKDKSKVAGYI
201 EIPDADIKEPVYPGPATREQLNRGVSFAEENESLDDQNIS
241 IAGHTFIDRPNYQFTNLKAAKKGSMVYFKVGNETRKYKMT
281 SIRNVKPTAVGVLDEQKGKDKQLTLITCDDYNEETGVWET
321 RKIFVATEVK

4 Kinetic Analysis Using Rate-Determining Approximation (Figures S19–S23, Equations S1–S11, and Table S1)

For the catalyzed cascade, experimentally, we cannot distinguish **B** and $\mathbf{I1}_c$, or $\mathbf{P}_c$ and **P**, on SDS-PAGE to study their transition kinetics. As previously mentioned in manuscript, the SrtA-mediated transition from $\mathbf{I2}_s$ to $\mathbf{P}_c$ is much faster than that the reaction between $\mathbf{I1}_c$ and **A**. Hence, we consider the latter as the rate-determining step and describe the overall reaction rate of catalyzed cascade as Equation S1:

$$v_{\text{cat-calcd.}} = k_{\text{AB(c)}}[\text{I1}_\text{c}][\text{A}] \qquad \text{(Equation S1)}$$

where $v_{\text{cat-calcd.}}$ is the calculated rate of catalyzed cascade by rate-determining step approximation, $k_{\text{AB(c)}}$ is the rate constant of the reaction between $\mathbf{I1}_c$ and **A**. To correlate with experimentally measurable parameters, we derive and express $[\text{I1}_\text{c}]$ as Equation S2:

$$[\text{I1}_\text{c}] = \frac{K_{\text{a(c)}}[\text{C}]_0 + K_{\text{a(c)}}[\text{A}] + 1 - \sqrt{\left(K_{\text{a(c)}}[\text{C}]_0 + K_{\text{a(c)}}[\text{A}] + 1\right)^2 - 4K_{\text{a(c)}}{}^2[\text{A}][\text{C}]_0}}{2K_{\text{a(c)}}} \qquad \text{(Equation S2)}$$

where $K_{\text{a(c)}}$ is the binding constant between CaM-SrtA and **B**, $[\text{C}_0]$ is the initial concentration of CaM-SrtA. We determined the value of $k_{\text{AB(c)}}$ as 90 $\text{M}^{-1}\text{s}^{-1}$ using the model reaction between SpyCatcher-M13 and GGG-SpyTag-YFP (Figures S19,S20). As previously discussed, $K_{\text{a(c)}}$ between CaM and **B** is 4.8×10^5 M^{-1}. This value can change based on different CaM•M13 pairs (see Table S1). Using these experimental data, we can deduce the theoretical kinetics and verify the validity of the model.

The presence of a rate-determining step implies that the system can reach steady state for certain period of time during reaction. We calculated the change of $[\text{I1}_\text{c}]$ with yield at 0.1 equivalent of CaM-SrtA and different initial concentrations of **A** ($[\text{A}_0]$) for $K_{\text{a(c)}} = 4.8 \times 10^5$ M^{-1} (Figure S21a). In the early stage (yield >40%), there is only a slight decrease of $[\text{I1}_\text{c}]$ with increasing yield, especially for high $[\text{A}_0]$. For example, $[\text{I1}_\text{c}]$ varies within ±6% at $[\text{A}_0]$= 10 μM, which can be regarded largely as a constant (0.77 ± 0.05 μM). The catalyzed cascade can thus be fitted with a first-order kinetic according to Equation S1 with an apparent rate constant $k_{\text{cat-exp.}}$ of (5.3 ± 0.6)× 10^{-5}s^{-1} (Figure S7f), and the calculated rate constant is written as Equation S3. The situation is similar for the system with M13-2 (Figure 21b). We also determined the $k_{\text{cat-calcd.}}$ at various $[\text{A}_0]$ for the system of M13-1 (Figure S21c) via Equation S3. The results agree well with experimental values (Figures S22–S23) with only slight discrepancy attributable to side reactions, especially at higher $[\text{A}_0]$. We further calculated $k_{\text{cat-calcd.}}$ with a wide range of $K_{\text{a(c)}}$ values (black curve in Figure S21d), which match well with experimentally determined values. The agreement confirms again the first-order kinetics of the catalyzed cascade.

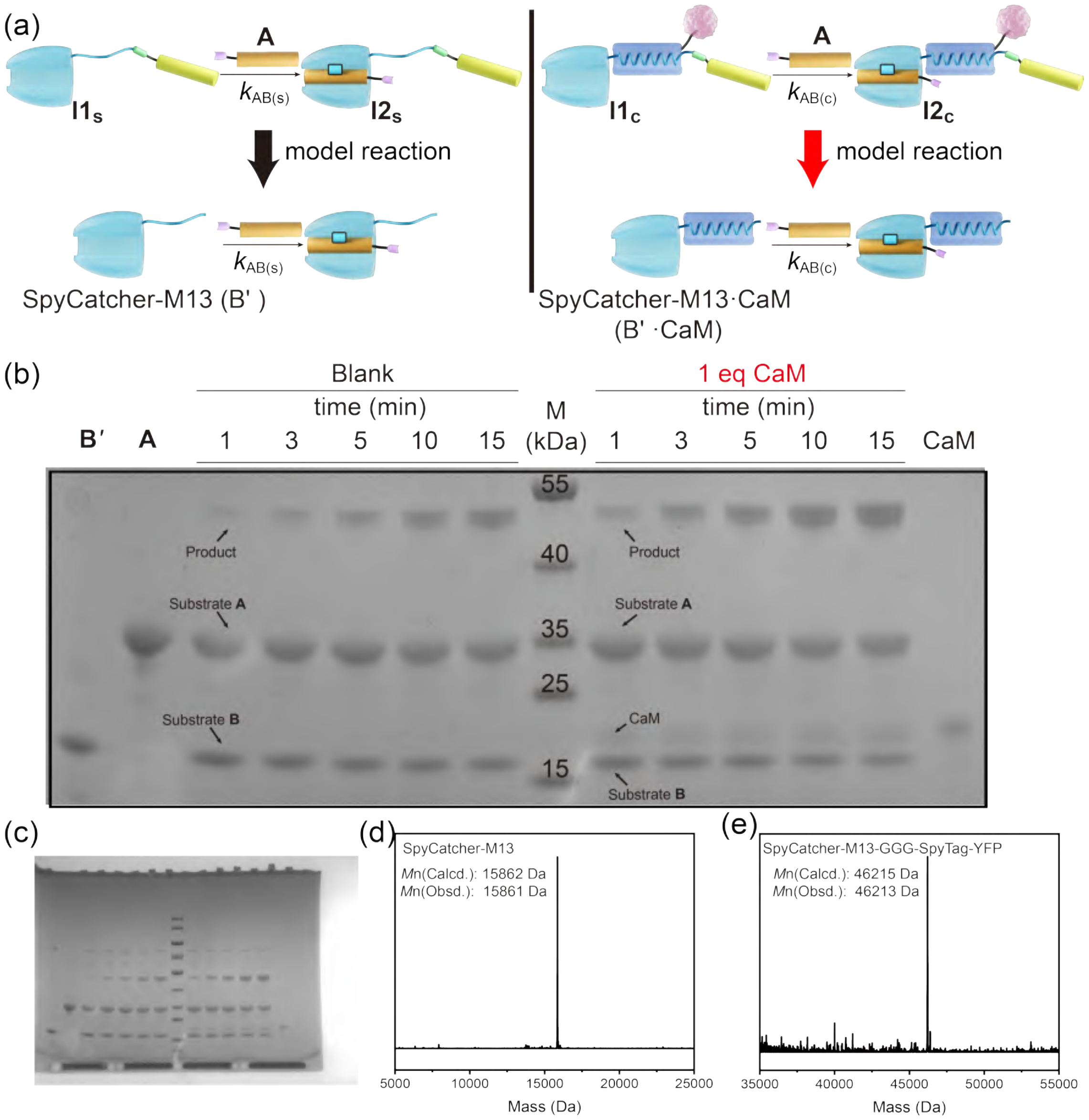

Figure S19: Investigating the kinetics of the reaction between SpyCatcher-M13 (**B′**) and GGG-SpyTag-YFP (**A**). **(a)** Model reactions to obtain $k_{AB(s)}$ and $k_{AB(c)}$. **(b)** Investigating the kinetics by SDS-PAGE (cropped gel). In all experiments, **A** and **B′** were mixed in 1:1 ratio at 50 μM. (Buffer condition: 25 mM Tris-HCl, 500 mM NaCl, 10 mM $CaCl_2$, pH 8.0) **(c)** Uncropped gel for Figure S19b. **(d)** MS spectra of SpyCatcher-M13. **(e)** MS spectra of SpyCatcher-M13-GGG-SpyTag-YFP.

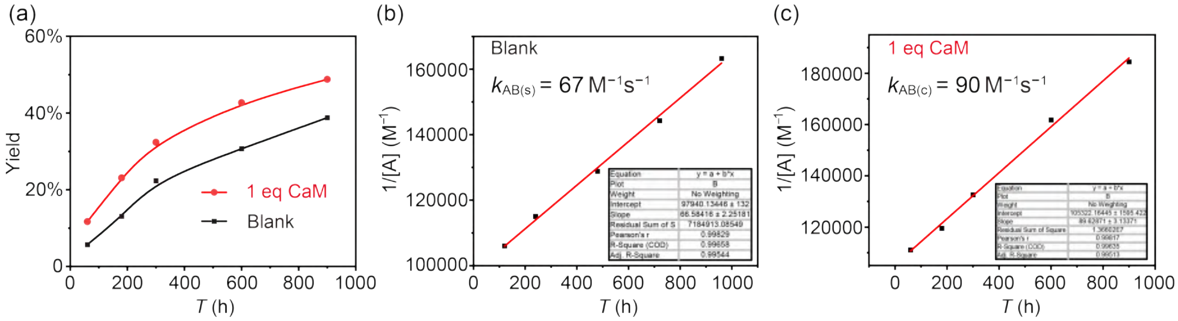

Figure S20: Kinetic results of the reaction between SpyCatcher-M13 (**B′**) and GGG-SpyTag-YFP (**A**). **(a)** Kinetic profiles extracted from Figure S19c. Addition of 1 equivalent CaM can slightly accelerate the reaction rate between **B′** and **A**, probably because the binding of CaM and M13 can reduce the steric effect caused by the flexible M13 and facility the capture of SpyTag by SpyCatcher. **(b,c)** Linear fitting of the kinetic data extracted from Figure S20a using second order kinetic model.

$$k_{\text{cat-calcd.}} = k_{\text{AB(c)}}[\text{I1}_\text{c}]$$
$$= k_{\text{AB(c)}} \frac{K_{\text{a(c)}}[\text{C}]_0 + K_{\text{a(c)}}[\text{A}] + 1 - \sqrt{\left(K_{\text{a(c)}}[\text{C}]_0 + K_{\text{a(c)}}[\text{A}] + 1\right)^2 - 4K_{\text{a(c)}}{}^2[\text{A}][\text{C}]_0}}{2K_{\text{a(c)}}} \quad \text{(Equation S3)}$$

Kinetic equation of spontaneous cascade can be similarly derived, assuming the reaction between $\mathbf{I1}_\text{s}$ and **A** as the rate-determining step. Again, we cannot distinguish **B** and $\mathbf{I1}_\text{s}$, or $\mathbf{P}_\text{s}$ and **P**, on SDS-PAGE. Assuming that **B** and $\mathbf{I1}_\text{s}$ always exist in an equilibrium, the concentration of $\mathbf{I1}_\text{s}$ ($[\text{I1}_\text{s}]$) can be obtained using Equation S4.

$$K_{\text{a(s)}} = \frac{[\text{I1}_\text{s}]}{[\text{B}]} \quad \text{(Equation S4)}$$

Moreover, as SpyCatcher has a medium binding affinity with SpyTag(DA) ($K_\text{d}{\sim}10^{-6}$ M)[7], $K_{\text{a(s)}}$ is supposed to be much smaller than 1 (simulated value can be found in Table 1). Hence, [B] can be regarded roughly as the same value with [A], as illustrated in Equation S5.

$$[\text{A}] = [\text{B}] + [\text{I1}_\text{s}] = [\text{B}](1 + K_{\text{a(s)}}) \approx [\text{B}] \quad \text{(Equation S5)}$$

With these assumptions, the kinetic equation of spontaneous cascade can be expressed as Equation S6.

$$v_{\text{spont}} = k_{\text{AB(s)}}[\text{I1}_\text{s}][\text{A}] = k_{\text{AB(s)}}K_{\text{a(s)}}[\text{B}][\text{A}] \approx k_{\text{spont}}[\text{A}]^2 \quad \text{(Equation S6)}$$

where v_{self} is the rate of spontaneous cascade by rate-determining approximation, $k_{\text{AB(s)}}$ is the rate constant of the reaction between $\mathbf{I1}_\text{s}$and **A**, and $K_{\text{a(s)}}$ is the equilibrium constant of **B** to $\mathbf{I1}_\text{s}$.

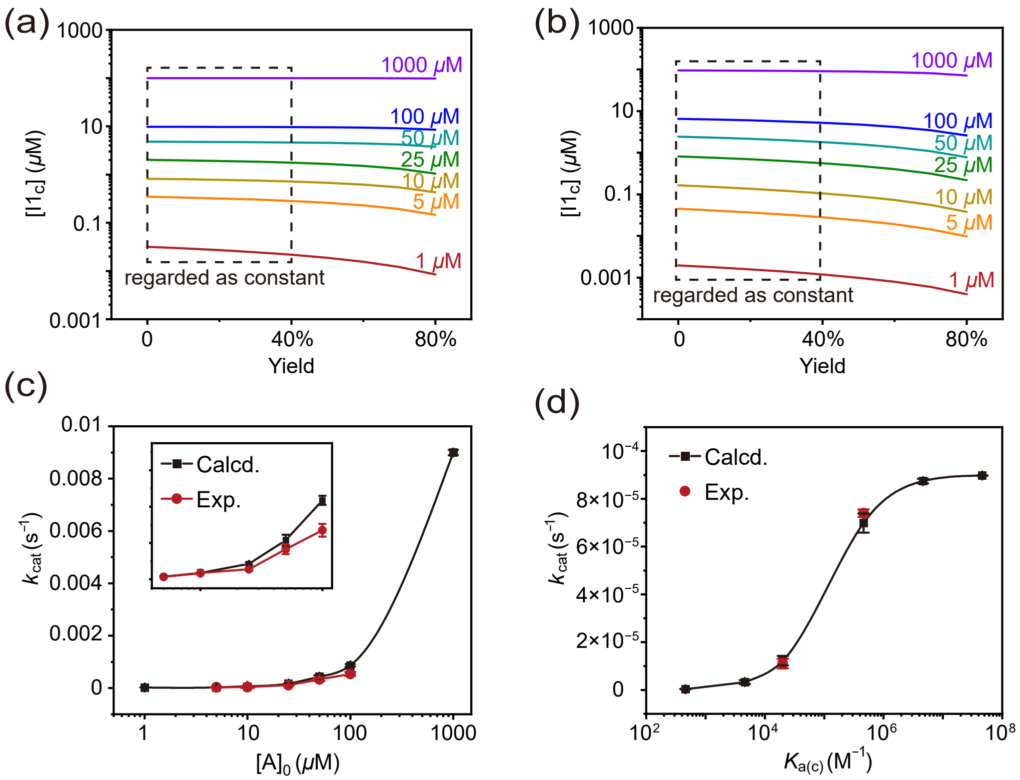

Figure S21: Calculated kinetics based on rate-determining approximation. **(a)** Calculated profiles of [I1$_c$] *versus* yield at $K_{a(c)} = 4.8\times10^5$ M^{-1} and 0.1 equivalent of CaM-SrtA. **(b)** Calculated profiles of [I1$_c$] *versus* yield at $K_{a(c)} = 2.0 \times 10^4$ M^{-1} and 0.1 equivalent of CaM-SrtA. **(c)** Concentration-dependent rate constants k_{cat} calculated via Equation S3 (black) and experimentally obtained values (red) for catalyzed cascade using first-order kinetic model at $K_{a(c)} = 4.8 \times 10^5$ M^{-1} and 0.1 equivalent of CaM-SrtA. **(d)** $K_{a(c)}$-dependent k_{cat} profile (black) calculated via Equation S3 and experimentally obtained values (red) using first-order kinetic model at $[A_0] = [B_0] = 10$ μM and 0.1 equivalent of CaM-SrtA.

Therefore, spontaneous cascade follows a second order kinetics and the rate constant k_{spont} can be calculated as follows:

$$k_{spont} = k_{AB(s)} K_{a(s)} \quad \text{(Equation S7)}$$

We determine the value of $k_{AB(s)}$ as 67 M^{-1}s^{-1} using the model reaction between SpyCatcher-M13 and GGG-SpyTag-YFP (Figures S20). The kinetic data of spontaneous cascade can thus be calculated accordingly (Table S1). Similar k_{spont} values, about 0.4 M^{-1}s^{-1}, were obtained when using different M13 sequences, indicating the variation of M13 sequence did not change the assembly-reaction profile. To quantify the rate enhancement in catalyzed cascade, we calculated

the initial rate enhancement ($v_{0(\text{cat})}/v_{0(\text{spont})}$) and the corresponding half-life times ($t_{1/2}$). For M13-1, there is a 12-fold increase in initial reaction rate and more than 17-fold reduction in $t_{1/2}$ with only 0.1 equivalent of CaM-SrtA. The rate enhancement gets lower with lower binding affinity of CaM•M13-2 and is completely lost in CaM•M13-3, which indicates the critical role of $K_{\text{a(c)}}$ for catalysis. An enhanced binding affinity typically correlates with a higher concentration of $\mathbf{I1}_{\text{c}}$, thereby accelerating the assembly-reaction cascade. Although this rate-determining approximation captures the essence of acceleration, we need a more comprehensive kinetic model to understand the process thoroughly and reveal the influence of specific parameters on catalyzed cascade in detail.

The concentration of $\mathbf{I1}_{\text{c}}$ ([I1$_{\text{c}}$]) is calculated as follows:

Because the two substrates **A** and **B** are mixed in 1:1 ratio and $\mathbf{I1}_{\text{c}}$ and **A** are consumed in 1:1 ratio, the relationship among [A], [B] and [I1$_{\text{c}}$] can be described as Equation S8.

$$[\text{A}] = [\text{B}] + [\text{I1}_\text{c}] \qquad \text{(Equation S8)}$$

Because $\mathbf{I2}_{\text{c}}$ will be consumed quickly once it is formed, and the dissociation of $\mathbf{P}_{\text{c}}$ into **C** and **P** is also assumed to be a fast step, the total amount of **C** ($[\text{C}]_0$) can be described as Equation S9.

$$[\text{C}]_0 = [\text{C}] + [\text{I1}_\text{c}] \qquad \text{(Equation S9)}$$

The relationship between [B] and [C] can further be calculated according to Equation S10.

$$K_{\text{a(c)}} = \frac{[\text{I1}_\text{c}]}{[\text{B}][\text{C}]} \qquad \text{(Equation S10)}$$

Then the description of [I1$_{\text{c}}$] can be obtained through combining Equations S8–S10 and shown as Equation S11 (Equation S2)

$$[\text{I1}_\text{c}] = \frac{K_{\text{a(c)}}[\text{C}]_0 + K_{\text{a(c)}}[\text{A}] + 1 - \sqrt{\left(K_{\text{a(c)}}[\text{C}]_0 + K_{\text{a(c)}}[\text{A}] + 1\right)^2 - 4K_{\text{a(c)}}{}^2[\text{A}][\text{C}]_0}}{2K_{\text{a(c)}}} \qquad \text{(Equation S11)}$$

Table S1: Summary of kinetic parameters governing the catalytic efficiency.

	M13-1	M13-2	M13-3
sequence	LRRGQILWNLGLNRIQTQIK	INLKALAALAQIIL	NLGLNRIQTQIK
$K_{a(c)}$ (M^{-1})	4.8×10^5	2.0×10^4	−−
$k_{\text{cat-exp.}}$ (s^{-1})^a	$(5.3 \pm 0.6) \times 10^{-5}$	$(1.1 \pm 0.2) \times 10^{-5}$	−−
$k_{\text{cat-calcd.}}$ (s^{-1})^b	$(6.9 \pm 0.4) \times 10^{-5}$	$(1.3 \pm 0.2) \times 10^{-5}$	−−
k_{spont} ($M^{-1}s^{-1}$)	0.46 ± 0.04	0.45 ± 0.05	0.34 ± 0.05
$v_{0(\text{cat-exp.})}$ (M s^{-1})^c	$(5.3 \pm 0.6) \times 10^{-10}$	$(1.1 \pm 0.2) \times 10^{-10}$	−−
$v_{0(\text{spont})}$ (M s^{-1})^d	$(4.6 \pm 0.4) \times 10^{-11}$	$(4.5 \pm 0.5) \times 10^{-11}$	$(3.4 \pm 0.5) \times 10^{-11}$
$v_{0(\text{cat-exp.})}/v_{0(\text{spont})}$	12	2.4	−−
$t_{1/2(\text{cat-exp.})}$ (h)^e	3.6 ± 0.3	17 ± 2	−−
$t_{1/2(\text{cat-calcd.})}$ (h)^f	2.8 ± 0.2	15 ± 2	−−
$t_{1/2(\text{spont})}$ (h)^g	60 ± 5	62 ± 7	87 ± 15
$t_{1/2(\text{spont})}/t_{1/2(\text{cat-exp.})}$	17	3.6	−−

[a]Experimentally obtained rate constant of catalyzed cascade(0.1 equivalent of CaM-SrtA, [A_0]= [B_0]= 10 μM).
[b]Calculated rate constant of catalyzed cascade via Equation S3 (0.1 equivalent of CaM-SrtA, [A_0]= [B_0]= 10 μM).
[c]Experimentally obtained initial rate of catalyzed cascade (0.1 equivalent of CaM-SrtA, [A_0]= [B_0]= 10 μM).
[d]Experimentally obtained initial rate of spontaneous cascade ([A_0]= [B_0]= 10 μM).
[e]Experimentally obtained half-life time of catalyzed cascade (0.1 equivalent of CaM-SrtA, [A_0]= [B_0]= 10 μM).
[f]Calculated half-life time of catalyzed cascade(0.1 equivalent of CaM-SrtA, [A_0]= [B_0]= 10 μM).
[g]Experimentally obtained half-life time of spontaneous cascade ([A_0]= [B_0]= 10 μM).

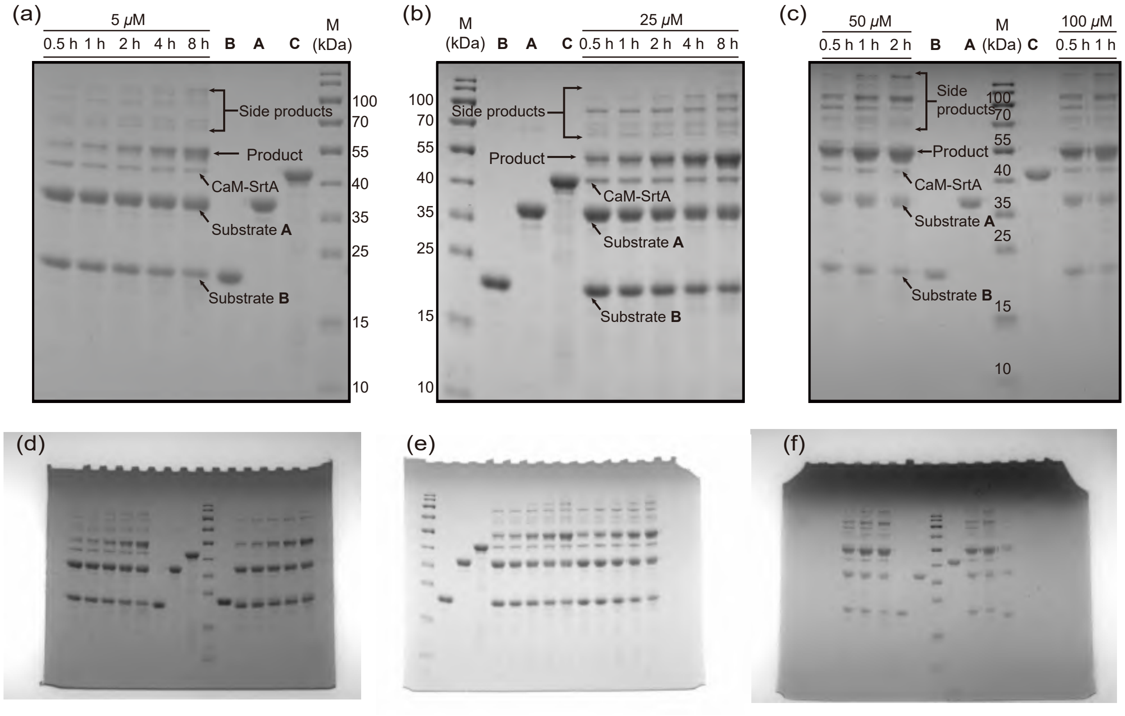

Figure S22: Concentration dependent experiments for catalyzed cascade. In all experiments, **A** and **B** were mixed in 1:1 ratio with the addition of 0.1 equivalent CaM-SrtA. **(a)** 5 μM (cropped gel). **(b)** 25 μM (cropped gel). **(c)** 50 μM and 100 μM (cropped gel). **(d)** Uncropped gel for Figure S22a. **(e)** Uncropped gel for Figure S22b. **(f)** Uncropped gel for Figure S22c. (Buffer condition: 25 mM Tris-HCl, 500 mM NaCl, 10 mM $CaCl_2$, pH 8.0)

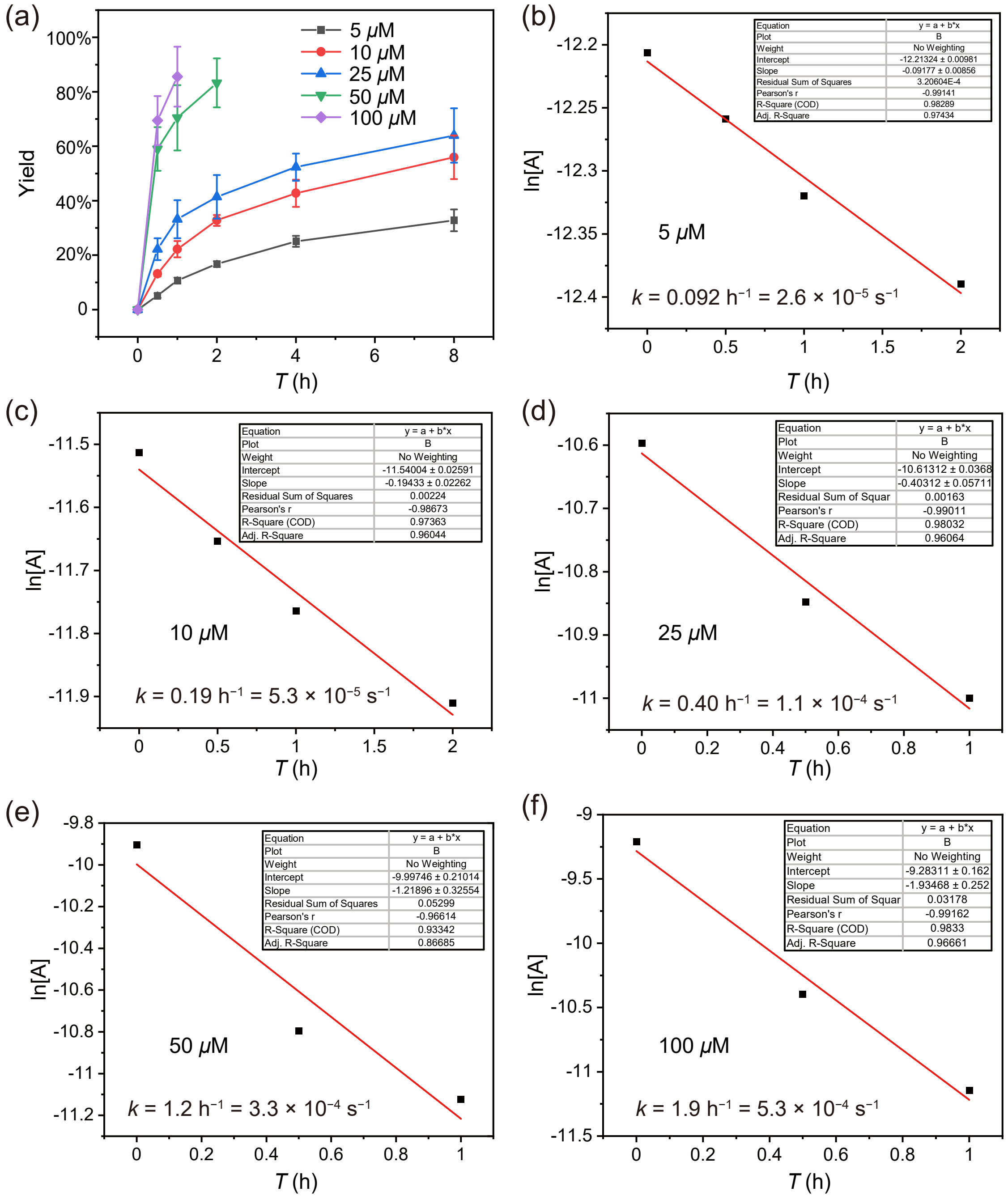

Figure S23: Concentration dependent experiments for catalyzed cascade. **(a)** Kinetic profiles extracted from Figure S22 and Figure 1e. **(b–f)** Linear fitting of the kinetic data extracted from Figure S23a using first order kinetic model.

5 Additional Discussions (Figures S24–S27)

Part A: Roles of CaM-SrtA in accelerating the assembly-reaction cascade

Firstly, the CaM domain in **C** binds to M13 peptides, triggering the coil-helix transition that aids in the dissociation between SpyCatcher and SpyTag(DA) in **B**, thereby promoting the covalent binding between SpyCacher and **A**. Secondly, the spatial proximity of CaM and SrtA in the catalyst may gifts SrtA with higher activity, facilitating the SrtA-mediated cleavage and ligation. Thirdly, the SrtA-mediated cyclization resets the M13 peptide to its loop conformation, preventing the reversion to coil-helix conformation and allowing CaM to dissociate spontaneous. Finally, the re-binding of CaM to **B** also disengages SrtA from the product, priming it for another cyclization.

Furthermore, although the binding constant of CaM to its substrate is relatively high (4.8×10^5 M^{-1}), the Gibbs free energy of binding ($\Delta G \approx -6.0$ kcal/mol) does not counteract the energetic drive for peptide bond formation mediated by SrtA. The Gibbs free energy associated with peptide bond formation is generally in the range of −15 to −20 kcal/mol, a significant difference that ensures that the SrtA-mediated ligation overcomes the energy barrier imposed by CaM dissociation. As a result, the overall cascade reaction proceeds efficiently, even in the presence of potential reversibility of the SrtA-mediated reactions.

Part B: Discussion on the reversibility of SrtA-mediated ligation

Although the SrtA-mediated ligation is inherently reversible, its efficiency can reach up to 95% when spatial conditions are favorable[8, 9]. In cases of single-substrate cyclization, the reversibility can vary significantly depending on the substrate structure, resulting in either high efficiency or relatively irreversible behavior[10, 11]. In our system, the reversibility of the ligation is essentially suppressed, and we propose several factors contributing to this phenomenon.

Firstly, the spatial proximity of CaM to SrtA within the fusion protein plays a crucial role in limiting the reversibility of the reaction. Once CaM dissociates from the product and binds to a new substrate, SrtA follows suit, effectively dissociating from the ligation. This ensures that SrtA is free to bind to new substrates, minimizing the likelihood of reverse binding events.

Secondly, the structural constraints imposed by the loop conformation of the M13 peptide hinder the re-association of SrtA. During cyclization, the M13 peptide adopts a loop structure that may shield the enzyme's active site. Once dissociated, SrtA may struggle to rebind the product due to these conformational restrictions. This loop structure thus acts as a protective "shield", further reducing the likelihood of reverse binding and effectively suppressing the ligation's reversibility.

Thus, while SrtA-mediated ligation inherently has a degree of reversibility, the design of our system-incorporating allosteric regulation by CaM, the structural protection provided by the M13

peptide loop, and the energetic driving forces-effectively minimizes this reversibility and promotes a successful, irreversible assembly-reaction cascade.

To further confirm that the reversibility of the ligation is indeed suppressed, we conducted an enzyme resistance assay. The product was incubated with SrtA enzyme at a 1:0.1 molar ratio, and samples were taken at regular intervals for mass spectrometry analysis (Figure S24a–c). The results revealed that, in the presence of 0.1 molar equivalent of SrtA, no product cleavage occurred over a 16-hour period. This observation indicates that, under the experimental conditions of this system, the SrtA-mediated ligation is effectively suppressed. Interestingly, even when the molar equivalent of SrtA was increased to 1, the product still remained stable over the 16-hour period (Figure S24d–f), further supporting the conclusion that the ligation's reversibility is significantly hindered in this system. Although the experiment in Figure S2 shows that the addition of 1 molar equivalent of CaM-SrtA leads to partial digestion of the protein, SrtA alone at the same concentration struggles to directly bind to the substrate. As a result, the product remains largely resistant to enzymatic cleavage. These findings are consistent with the structural constraints imposed by the M13 peptide loop, which acts as a protective shield, further minimizing the likelihood of reverse binding and preserving the stability of the assembly-reaction cascade.

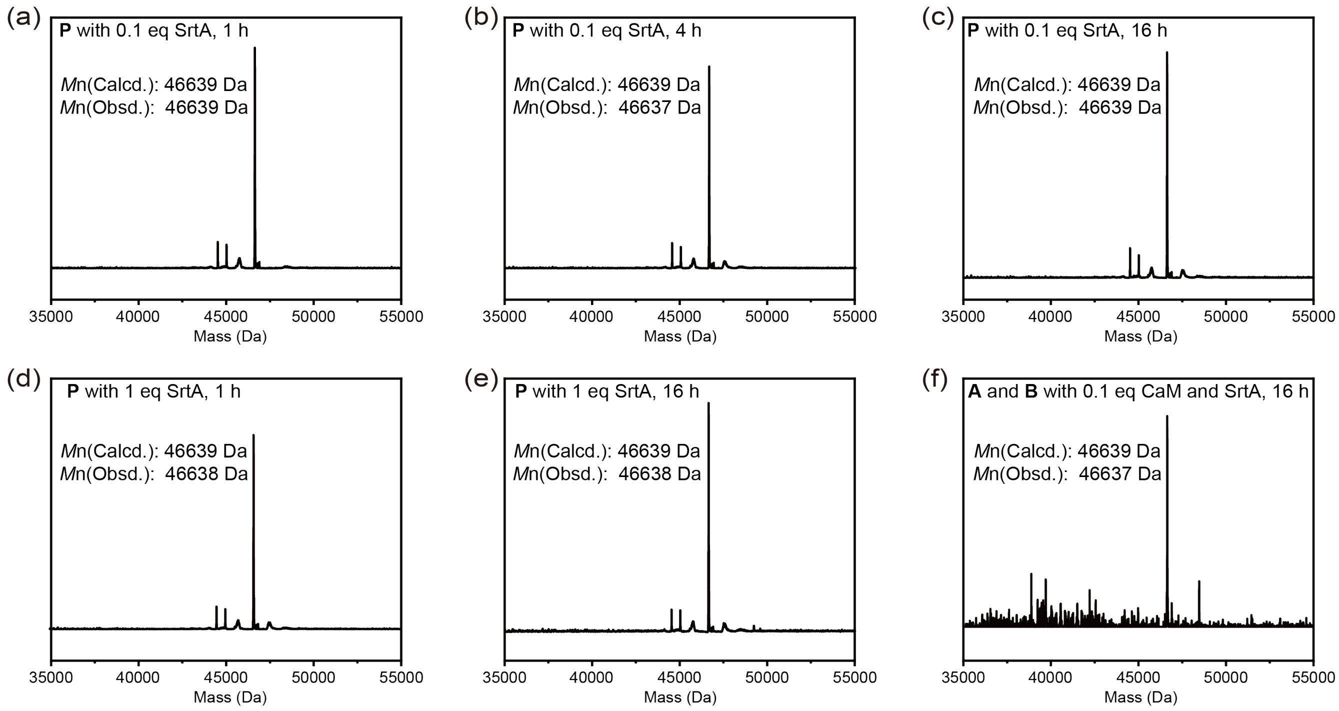

Figure S24: Experimental validation of the suppression of reversibility in SrtA-mediated ligation. **(a–c)** Mass spectrometry analysis of the product mixed with SrtA at a 1:0.1 molar ratio. Samples were taken at 1 h, 4 h, and 16 h, respectively. Only the product peak at 46639 Da was observed, with no indication of cleavage, suggesting effective suppression of the reversibility of the SrtA-mediated ligation. **(d,e)** Mass spectrometry analysis of the product mixed with SrtA at a 1:1 molar ratio. Samples were taken at 1 h and 16 h, respectively. Similarly, the product peak at 46639 Da remained intact, with no cleavage observed even at the higher SrtA concentration, further confirming the suppression of ligation reversibility in the system. **(f)** Enzyme activity validation experiment: **A** and **B** were mixed with 1:1 molar ratios, and 0.1 molar equivalents of both CaM and SrtA were added. After 16 hours, the mass spectrometry analysis showed the product peak at 46639 Da, confirming that the enzymes remain active under these conditions. Experimental condition: $[A_0]$= $[B_0]$= 10 μM, 25 mM Tris-HCl, 500 mM NaCl, 10 mM $CaCl_2$, pH 8.0.

Part C: Correlation and synergy between different scales

As catalytic behaviors concern kinetics, the importance of observation *time scale* is self-evident. We simulated the yield profile with different k_{AB} values at various times from 1 min to 48 h using the parameters in Table 1 (Figure S25a). This window of k_{AB} for considerable catalytic performance gradually shifts to lower values and becomes narrower as the observation time gets longer. If we plot $k_{\mathrm{AB}}T$ as the X-axis, all these curves collapse onto a composite curve, especially for the yield gain $\mathrm{Yield}_{(c)}/\mathrm{Yield}_{(s)}$, from which we can also define a window of $k_{\mathrm{AB}}T$ ($\sim 10^3$ to $\sim 10^4$ M^{-1}) for considerable catalytic performance (Figure S25b). It suggests that the two parameters are highly correlated and synergistic and that catalyzed cascade is meaningful either for faster reaction at shorter times, or for slower reaction at longer times. While k_{AB} values may vary from 10^{-3} $\mathrm{M}^{-1}\mathrm{s}^{-1}$ for common organic reactions[12] to $\sim 10^3$ $\mathrm{M}^{-1}\mathrm{s}^{-1}$ for click reactions or enzyme-catalyzed coupling reactions[13–15], the time frame may also vary from mins to hours correspondingly.

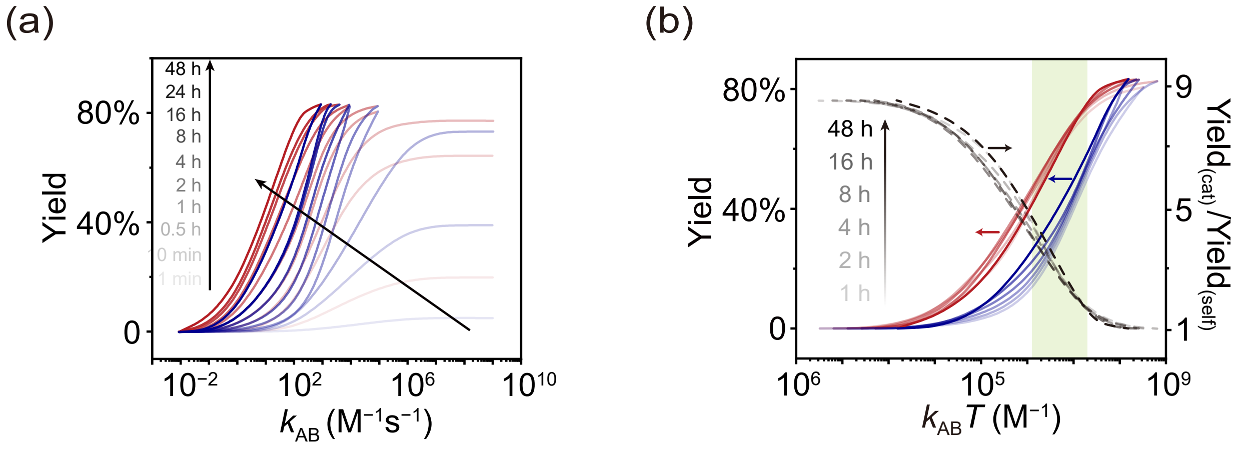

Figure S25: Correlation and synergy between k_{AB} and T(time). **(a)** Simulated profile of yield *versus* k_{AB} at different times from 1 min to 48 h. **(b)** Simulated profiles of yield versus $k_{\mathrm{AB}}T$ for both spontaneous and catalyzed cascades at various times. (0.1 equivalent of CaM-SrtA, $[\mathrm{A}]_0 = [\mathrm{B}]_0 = 10$ μM)

We also explored the influence of substrate concentration (Figure S26a,b), which represents the space scale relevant to catalyzed cascade. Under same $[\mathrm{A}]_0$ conditions, higher $K_{\mathrm{a(c)}}$ value brings higher $\mathrm{v}_{0(c)}/\mathrm{v}_{0(s)}$ value. Besides, the profile of $\mathrm{v}_{0(c)}/\mathrm{v}_{0(s)}$ to $[\mathrm{A}]_0$ seems to exhibit a plateau value for given $K_{\mathrm{a(c)}}$ values (Figure S26a). As the $K_{\mathrm{a(c)}}$ value increases, the threshold $[\mathrm{A}]_0$ value necessary to attain the plateau decreases correspondingly. We further plot the initial rate enhancement and yield in 8 h against $K_{\mathrm{a(c)}}[\mathrm{A}]_0$ for different $K_{\mathrm{a(c)}}$ values (Figure S26c,d). Interestingly, the curves for the initial rate enhancement $\mathrm{v}_{0(c)}/\mathrm{v}_{0(s)}$ collapse onto a composite curve (Figure S26c). This shows that *space scale* ($[\mathrm{A}]_0$)and *energy scale* ($K_{\mathrm{a(c)}}$) are strongly correlated. Although with a plateau,

the composite curve exhibits a monotonically increasing trend. Therefore, increasing $K_{a(c)}$ not only results in substantial catalytic behavior as shown by the higher rate enhancement, but also broadens the window of considerable catalytic performance towards higher $K_{a(c)}[A]_0$, meaning that catalyzed cascade can be applied to a broader range of substrate concentrations at higher $K_{a(c)}$ values.

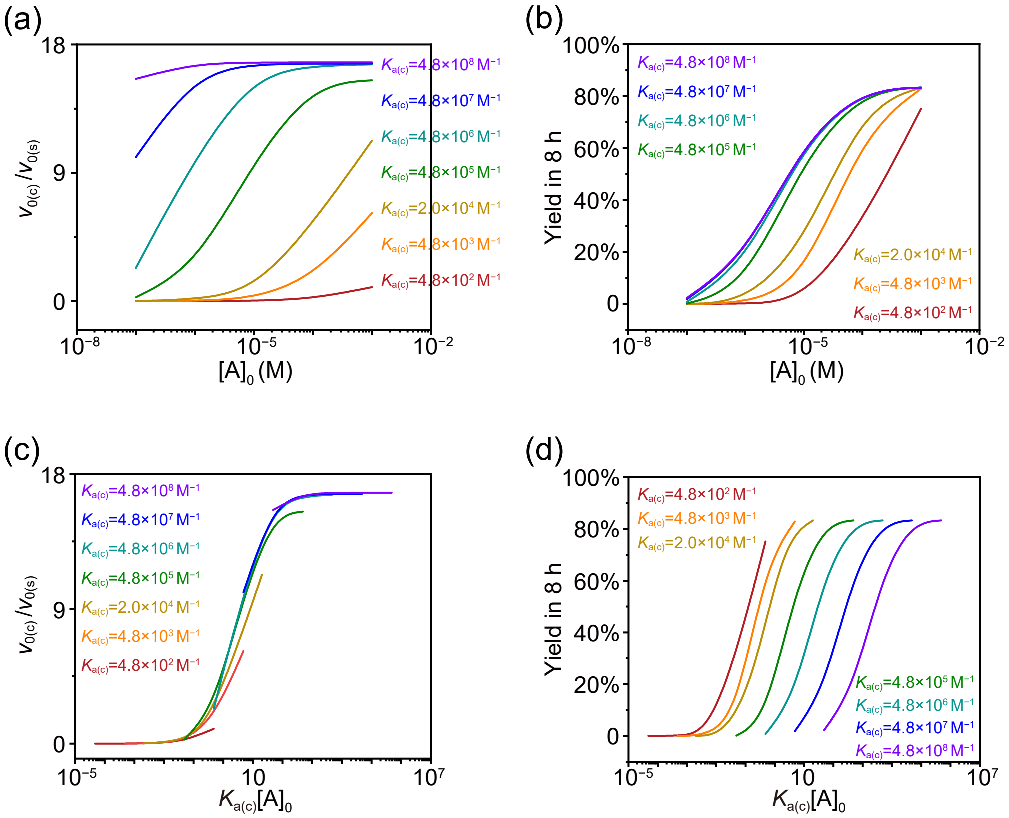

Figure S26: Synergy between $K_{a(c)}$ and $[A]_0$. **(a)** Simulated profile of $v_{0(c)}/v_{0(s)}$ *versus* $[A]_0$ at different $K_{a(c)}$. **(b)** Simulated profile of yield in 8 h *versus* $[A]_0$. **(c)** Simulated profile of $v_{0(cat)}/v_{0(self)}$ *versus* $K_{a(c)}[A]_0$. **(d)** Simulated profile of yield in 8 h *versus* $K_{a(c)}[A]_0$. (0.1 equivalent of CaM-SrtA, $[A]_0 = [B]_0 = 10\ \mu M$)

The above exploration indicates that for such complex systems, the influences of various parameters are highly correlated and often synergistic. Consequently, these factors often show equivalent influences. To exhibit considerable catalytic performance, the effective range of these parameters may actually span up to 3∼4 orders of magnitude on energy, time, and space scales. Although this is drawn from an artificial system, we assume that the underlying physical principles may apply to catalyzed assembly-reaction cascade a in biological systems as well.

Part D: Tuning catalysis for optimal performance

For the system shown in Scheme 1f, the catalyst loading was fixed at 0.1 equivalent which gave the best catalytic behavior in 8 h (Figure 1e). The maximum total turnover number (TON) is thus limited to be less than 10. To optimize the catalytic performance, we need a proper combination of parameters that generates satisfactory yield at relatively short times with a high TON. The influence of catalyst loading at different stoichiometry is confirmed by simulation (Figure S27a). While the yield in 8 h shows a maximum at 0.1 equivalent in good agreement with experiments, the initial rate enhancement shows a maximum at 1 equivalent. We inferred that CaM-M13 binding is critical for gate-opening and thus, the more CaM there is, the faster the $v_{0(\mathrm{cat})}$ is. As CaM and SrtA exist as a fusion protein, more CaM-SrtA loading also means more SrtA, which shall simultaneously promote the spontaneous cascade pathway as well as the side reactions. An optimal catalytic performance would require an extremely accelerated cascade with rapid gate-opening, covalent reaction and cyclization-induced detachment and an extremely retarded spontaneous cascade process with tight gating. The former mandates a large $K_{\mathrm{a(c)}}$, a moderate k_{AB}, a sufficiently fast k_{SrtA}, and a sufficiently large K_{d}; the latter demands a very small $K_{\mathrm{a(s)}}$ and a moderate k_{AB}. As shown in Scheme 1f, $K_{\mathrm{a(c)}}$ and $K_{\mathrm{a(s)}}$ are correlated by a factor of K_{CaM}. In literature, the smallest K_{CaM} was found to be $\sim10^{-9}$ M[16]. With directed evolution, we anticipated that a CaM-peptide pair with $\sim10^{-10}$ M may be experimentally accessible. The smaller K_{CaM} is, the better catalytic performance is. For SpyTag-SpyCatcher reaction, the third generation has a k_{AB} of $\sim10^5$ $\mathrm{M^{-1}s^{-1}}$, which is fast approaching the diffusion limit[17]. With mutation, it can vary between 0 and 10^5 $\mathrm{M^{-1}s^{-1}}$. For SrtA-mediated ligation, the rate constant of cyclization in literature is reported to be ~1 $\mathrm{s^{-1}}$[18]. We hypothesized the it may also achieve $\sim10^5$ $\mathrm{s^{-1}}$ similar to OaAEP in literature[19], with the help of directed evolution. Based on these information, we simulated that, in 8 h, the TON can achieve ∼10000 for a set of ideal parameters (Figure S30b, dashed line). In practice, we anticipate that a TON of ∼1000 is probably more attainable with a set of more realistic parameters (Figure S27b, solid line). This value is on par with the model catalyzed assembly system based on toehold-mediated strand displacement[20, 21]. The above result has important implication in that the parameters can be tuned individually to optimize the catalytic performance, which is probably regulated by directed evolution in biological systems.

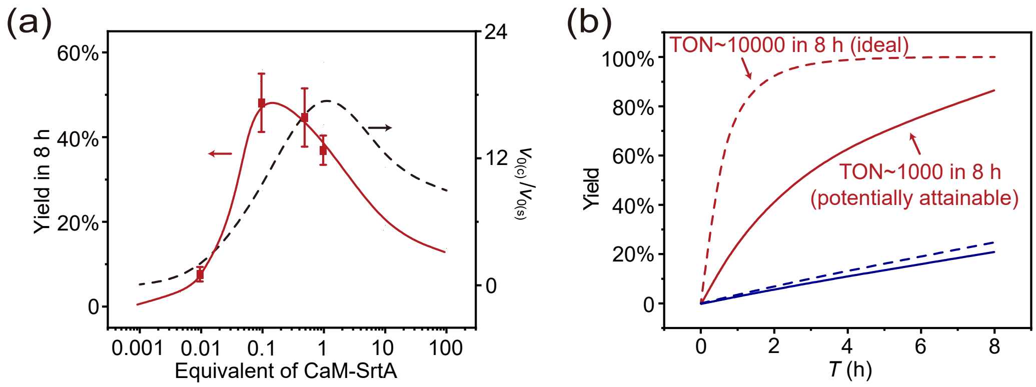

Figure S27: Optimizing the catalytic performance. **(a)** Simulated profiles of yield in 8 h (red line) and initial rate enhancement (black dashed line) versus equivalent of CaM-SrtA. The experimental data are shown as individual red points with error bar (3 repeated experiments). **(b)** Ideal kinetic profiles for spontaneous (blue dashed line) and catalyzed (red dashed line) cascades achieving a TON of ∼10000 in 8 h and potentially attainable kinetic profile for spontaneous (blue solid line) and catalyzed cascades (red solid line) achieving a TON of ∼1000 in 8 h. For the former, the ideal set of parameters is $K_{a(c)} = 10^8$ M^{-1}, $K_{a(s)} = 10^{-4}$, $K_d = 1$ M, $k_{AB} = 10^6$ M^{-1}s^{-1}, $k_{SrtA} = 10^5$ s^{-1}, $k_{side} = 10$ s^{-1}, 0.0001 equivalent of CaM-SrtA. For the latter, the potentially attainable set of parameters is $K_{a(c)} = 10^6$ M^{-1}, $K_{a(s)} = 10^{-4}$, $K_d = 1$ M, $k_{AB} = 5 \times 10^6$ M^{-1}s^{-1}, $k_{SrtA} = 10^4$, k_{side} $= 10$ s^{-1}, 0.001 equivalent of CaM-SrtA.

Supplementary References

[1] Brautigam, C. A.; Zhao, H.; Vargas, C.; Keller, S.; Schuck, P. Integration and Global Analysis of Isothermal Titration Calorimetry Data for Studying Macromolecular Interactions. *Nature Protocols* **2016**, *11*, 882–894.

[2] Jumper, J. et al. Highly Accurate Protein Structure Prediction With AlphaFold. *Nature* **2021**, *596*, 583–589.

[3] Row, R. D.; Roark, T. J.; Philip, M. C.; Perkins, L. L.; Antos, J. M. Enhancing the Efficiency of Sortase-Mediated Ligations Through Nickel-Peptide Complex Formation. *Chemical Communications* **2015**, *51*, 12548–12551.

[4] Wu, Z. M.; Guo, X. Q.; Guo, Z. W. Sortase A-Catalyzed Peptide Cyclization for the Synthesis of Macrocyclic Peptides and Glycopeptides. *Chemical Communications* **2011**, *47*, 9218–9220.

[5] Yamauchi, S.; Kobashigawa, Y.; Fukuda, N.; Teramoto, M.; Toyota, Y.; Liu, C.; Ikeguchi, Y.; Sato, T.; Sato, Y.; Kimura, H.; Masuda, T.; Ohtsuki, S.; Noi, K.; Ogura, T.; Morioka, H. Cyclization of Single-Chain Fv Antibodies Markedly Suppressed Their Characteristic Aggregation Mediated by Inter-Chain VH-VL Interactions. *Molecules* **2019**, *24*.

[6] Qu, Z. Y.; Fang, J.; Wang, Y. X.; Sun, Y. B.; Liu, Y. J.; Wu, W. H.; Zhang, W. B. A Single-Domain Green Fluorescent Protein Catenane. *Nature Communications* **2023**, *14*, 3480.

[7] Zakeri, B.; Fierer, J. O.; Celik, E.; Chittock, E. C.; Schwarz-Linek, U.; Moy, V. T.; Howarth, M. Peptide Tag Forming a Rapid Covalent Bond to a Protein, Through Engineering a Bacterial Adhesin. *Proceedings of the National Academy of Sciences of the United States of America* **2012**, *109*, E690–E697.

[8] Westerlund, K.; Myrhammar, A.; Tano, H.; Gestin, M.; Karlström, A. E. Stability Enhancement of a Dimeric HER2-Specific Affibody Molecule Through Sortase A-Catalyzed Head-to-Tail Cyclization. *Molecules* **2021**, *26*, 2874.

[9] Yang, M.; Hong, H. F.; Liu, S. Z.; Zhao, X. R.; Wu, Z. M. Immobilization of Staphylococcus Aureus Sortase A on Chitosan Particles and Its Applications in Peptide-to-Peptide Ligation and Peptide Cyclization. *Molecules* **2018**, *23*, 192.

[10] Row, R. D.; Roark, T. J.; Philip, M. C.; Perkins, L. L.; Antos, J. M. Enhancing the Efficiency of Sortase-Mediated Ligations Through Nickel-Peptide Complex Formation. *Chemical Communications* **2015**, *51*, 12548–12551.

[11] Wu, Z. M.; Guo, X. Q.; Guo, Z. W. Sortase A-Catalyzed Peptide Cyclization for the Synthesis of Macrocyclic Peptides and Glycopeptides. *Chemical Communications* **2011**, *47*, 9218–9220.

[12] Kim, E.; Koo, H. Biomedical Applications of Copper-Free Click Chemistry: In Vitro, in Vivo, and ex Vivo. *Chemical Science* **2019**, *10*, 7835–7851.

[13] McKay, C. S.; Finn, M. G. Click Chemistry in Complex Mixtures: Bioorthogonal Bioconjugation. *Chemistry & Biology* **2014**, *21*, 1075–1101.

[14] Cao, Y.; Liu, D.; Zhang, W.-B. Supercharging SpyCatcher Toward an Intrinsically Disordered Protein With Stimuli-Responsive Chemical Reactivity. *Chemical Communications* **2017**, *53*, 8830–8833.

[15] Yang, T.; Liu, Y.; Wu, W.-H.; Peng, R.; Zhang, W.-B. A Moonlighting Superpositively Charged SpyCatcher. *CCS Chemistry* **2023**, *5*, 2663–2673.

[16] Corbacho, I.; Berrocal, M.; Török, K.; Mata, A. M.; Gutierrez-Merino, C. High Affinity Binding of Amyloid β-Peptide to Calmodulin: Structural and Functional Implications. *Biochemical and Biophysical Research Communications* **2017**, *486*, 992–997.

[17] Zhou, H. X.; Pang, X. D. Electrostatic Interactions in Protein Structure, Folding, Binding, and Condensation. *Chemical Reviews* **2018**, *118*, 1691–1741.

[18] Antos, J. M.; Popp, M. W. L.; Ernst, R.; Chew, G. L.; Spooner, E.; Ploegh, H. L. A Straight Path to Circular Proteins. *Journal of Biological Chemistry* **2009**, *284*, 16028–16036.

[19] Morgan, H. E.; Turnbull, W. B.; Webb, M. E. Challenges in the Use of Sortase and Other Peptide Ligases for Site-Specific Protein Modification. *Chemical Society Reviews* **2022**, *51*, 4121–4145.

[20] Zhang, D. Y.; Winfree, E. Robustness and Modularity Properties of a Non-Covalent DNA Catalytic Reaction. *Nucleic Acids Research* **2010**, *38*, 4182–4197.

[21] Wu, Y. Q.; Zhang, D. Y.; Yin, P.; Vollmer, F. Ultraspecific and Highly Sensitive Nucleic Acid Detection by Integrating a DNA Catalytic Network With a Label-Free Microcavity. *Small* **2014**, *10*, 2067–2076.
